# Correction: A re-evaluation of silk measurement by the cecropia caterpillar (*Hyalophora cecropia*) during cocoon construction reveals use of a silk odometer that is temporally regulated

**DOI:** 10.1371/journal.pone.0230597

**Published:** 2020-03-12

**Authors:** The PLOS ONE Staff

## Notice of Republication

This article was republished on February 26, 2020, to correct errors introduced during the typesetting process in the captions for Figs 1 and 6 as well as in the Results, Discussion, and Materials and Methods sections. The publisher apologizes for these errors. Please download this article again to view the correct version. The originally published, uncorrected article and the republished, corrected article are provided here for reference.

## Supporting information

S1 File Originally published, uncorrected article. (PDF)

S2 File Republished, corrected article. (PDF)

## References

[1] Sehadova H, Guerra PA, Sauman I, Reppert SM (2020) A re-evaluation of silk measurement by the cecropia caterpillar (*Hyalophora cecropia*) during cocoon construction reveals use of a silk odometer that is temporally regulated. PLoS ONE 15(2): e0228453 10.1371/journal.pone.0228453 32074121 PMC7029867

