# Supplementary material for: A re-evaluation of silk measurement by the cecropia caterpillar (Hyalophora cecropia) during cocoon construction reveals use of a silk odometer that is temporally regulated
Source: PLoS One. 2020 Feb 19;15(2):e0228453. doi: 10.1371/journal.pone.0228453 (PMC7029867; doi:10.1371/journal.pone.0228453)
Supplement: S1 Text — (PDF) [file pone.0228453.s001.pdf]

S1 Text: Spinneret details:

From the dorsal view, the posterior region of the silk press is characterized by a crescent-shaped cross-sectional depression that continues anteriorly through the silk press midline, forming on its either side a prominent valley to which dorsal and ventrolateral muscle fibers are attached (**S2 Fig, Fig 4**). In total, three pairs of muscles are attached to the cecropia silk press. The majority of dorsal muscle fibers in the anterior and middle silk press originate in a pair of robust anterior dorsal muscles. A pair of thinner posterior dorsal muscles is attached to the posterior silk press, just between the joint point of the silk gland and the crescent-shaped depression. Muscle fibers that are bound to the dorsolateral region of the anterior and middle silk press are part of paired ventral muscles that arise from the ventrolateral area of the head capsule. A pair of bilateral connectives rises directly from the joint point of the silk glands and runs in a ventrolateral direction, anchoring muscles running alongside each silk gland.

From the semi-thin cross-sections of the posterior silk press (**S3 Fig**), it is clear that the common duct changes its circular character into the thickened crescent-shaped profile that becomes wider on the sections from the anterior region. Near to the spigot entrance it has a nearly perfect ovular shape. The changes in the duct profile are in agreement with the shape of depression observed on the dorsal side of the silk press. In these sections, it is clear that the central part of the anterior-posterior depression of the silk press is formed by a thin septum and that the muscles are attached by the ligamentous connectives to the anterior ridges of the cuticular intima.
