# Supplementary material for: A re-evaluation of silk measurement by the cecropia caterpillar (Hyalophora cecropia) during cocoon construction reveals use of a silk odometer that is temporally regulated
Source: PLoS One. 2020 Feb 19;15(2):e0228453. doi: 10.1371/journal.pone.0228453 (PMC7029867; doi:10.1371/journal.pone.0228453)
Supplement: S2 Text — (PDF) [file pone.0228453.s002.pdf]

S2 Text: Spinneret comparison with other species:

A comparison of the available data that describes the overall morphology of silk glands revealed considerable differences between their arrangements within the body plan of lepidopteran larvae. Most lepidopteran species show a similar organization of the silk gland, that is characterized by a very thin anterior and posterior region, and by a thick middle region with two characteristic bends that delineate three loops laid above one another. However, the relative size of these three major silk gland compartments, as well as the bending pattern of the posterior silk gland region, differs significantly among lepidopteran species [1]. While the caterpillar silk glands of *Pieris rapae*, *Barathrea brussicae*, and *Helicoverpa armigera* have straight posterior parts, the caterpillar silk glands in the wax moth, *Galleria mellonella,* and the domesticated silkmoth, *Bombyx mori*, possess many loops. Nevertheless, *G. mellonella* has relatively smaller posterior and relatively larger middle silk gland regions, as compared to *B. mori* [2-4]. In *Euproctis flava,* the length of the posterior region is dramatically reduced.

The morphology of the silk glands in the larvae of some saturniid moths (*Antheraea yamamai* and *Antheraea pernyi)* also differs significantly from the species mentioned above [2,5]. Their glands are typically organized as tightly arranged high amplitude loops that are recumbent in the dorso-ventral direction, and that lie along almost the entire body length of the caterpillar. Such an arrangement allows the gland to be almost five times longer than the entire length of the larval body.

The general layout and anatomy of the silk glands of *H. cecropia* larvae resembles the one observed in closely related saturniid species, *A. pernyi* and *A. yamamai.* The silk gland consists of a tube of polyploid secretory cells that embrace the inner lumen of the gland. The cells are typically arranged in two rows (so called "duplex type"). However, in some rare cases (i.e. caterpillars of *Psychidae* and *Lymanttridae*), there are more than two rows of silk gland secretory cells (the "polyplex type") [2]. Although the anterior silk gland in most studied species has a duplex arrangement of cells with typically branched polyploid nuclei, this part of the gland of the wax moth, *G. mellonella,* is composed of duplexes containing very elongated cells with polyploid cylindrical nuclei without any marks of ramification [4]. Proximally from the merged point of the anterior regions of paired glands, there is the outfall of accessory glands called Lyonnet´s or Filippi´s glands. The Filippi's gland consists mostly of only a few voluminous cells that may differ in size and shape within *Lepidoptera* [6,7]. Although it was suggested that their

secretory product provides lubrication or a cement coating of the silk, the removal of the glands has no effect on the silk quality [8].

The detailed morphology of the silk press has so far been described in only a few lepidopteran species, i.e., *Helicoverpa armigera* and *B. mori* [3,9,10]. In these species, the 3D model of the silk press has been reconstructed based on subsequent histological sections. By comparing the single histological sections with those we obtained in cecropia, as well as the optical 3D reconstruction from these other species, we can conclude that the shape of the silk press, as well as the composition of all the attached muscles, are basically identical.

REFERENCES

1. Sehnal F, Akai H. Insect silk glands: their types, development and function, and effects of environmental factors and morphogenetic hormones on them. Int J Insect Morphol Embryol. 1990;19:79–132.
2. Akai H. *Ultrastructural Morphology of Insects.* (University of Tokyo Press, Tokyo, 1976).
3. Sorensen GS, Cribb BW, Merritt D, Johnson M-L, Zalucki MP. Structure and ultrastructure of the silk glands and spinneret of *Helicoverpa armigera* (Hübner) (Lepidoptera: Noctuidae). Arthropod Struct Dev. 2006;35:3–13.
4. Kludkiewicz B. et al. The expansion of genes encoding soluble silk components in the greater wax moth, *Galleria mellonella*. Insect Biochem Mol Biol. 2019:106;28-38.
5. Zurovec M. et al. Sericin composition in the silk of *Antheraea yamamai*. Biomacromolecules. 2016;17:1776–1787.
6. Victoriano E, Grego´rio EA. Ultrastructure of the Lyonet's glands in larvae of *Diatraea saccharalis fabricius* (Lepidoptera: Pyralidae). Biocell. 2004;28:165–169.
7. Patra S, Singh RN, Raziuddin M. Morphology and histology of Lyonet's gland of the tropical tasar silkworm, *Aantheraea mylitta*. J. Insect Sci. 2012;12:123.
8. Machida Y. Studies on the silk glands of the silkworm, *Bombyx mori* li morphological and functional studies of Filippi's glands in the silkworm. Science Bulletin of the Faculty of Agriculture, Kyushu University. 1965;22:95–108.
9. Magoshi J, Magoshi Y, Nakamura S. Mechanism of fiber formation of silkworm. Silk Polymers ACS Symposium Series. 1994;533:292–310.

10. Asakura T, Umemura K, Nakazawa Y, Hirose H, Higham J, Knight D. Some Observations on the Structure and Function of the Spinning Apparatus in the Silkworm *Bombyx mori*. *Biomacromolecules.* 2007;8**:**175–181.
