# Supplementary material for: A re-evaluation of silk measurement by the cecropia caterpillar (Hyalophora cecropia) during cocoon construction reveals use of a silk odometer that is temporally regulated
Source: PLoS One. 2020 Feb 19;15(2):e0228453. doi: 10.1371/journal.pone.0228453 (PMC7029867; doi:10.1371/journal.pone.0228453)
Supplement: S1 Fig — (PDF) [file pone.0228453.s003.pdf]

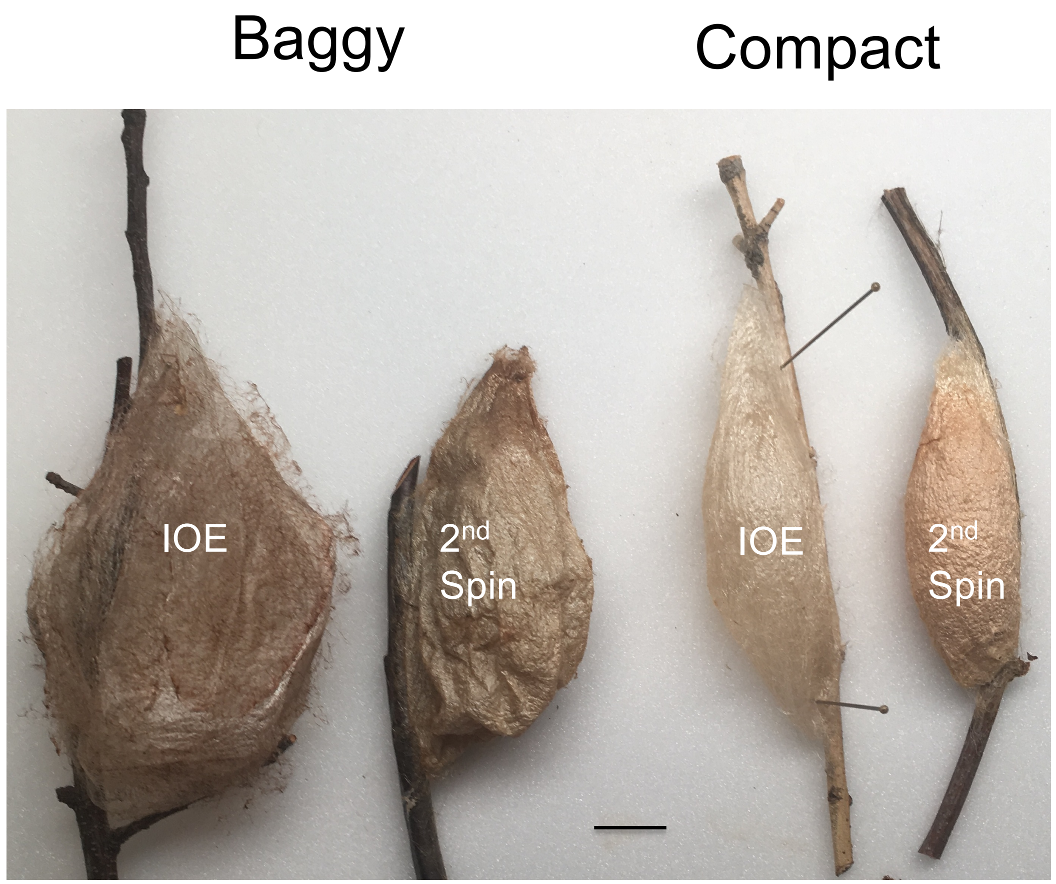

S1 Fig. Interrupting silk deposition during baggy cocoon construction. Examples of the initial outer envelope (IOE) and complete cocoon (2nd Spin) for Baggy (left) and Compact (right) cocoons. For each cocoon type, 2nd spin cocoons consist of a completed outer envelope, an intermediate space filled with silk, and an inner envelope. Scale bars = 1 cm.
