# Supplementary material for: A re-evaluation of silk measurement by the cecropia caterpillar (Hyalophora cecropia) during cocoon construction reveals use of a silk odometer that is temporally regulated
Source: PLoS One. 2020 Feb 19;15(2):e0228453. doi: 10.1371/journal.pone.0228453 (PMC7029867; doi:10.1371/journal.pone.0228453)
Supplement: S2 Fig — (PDF) [file pone.0228453.s004.pdf]

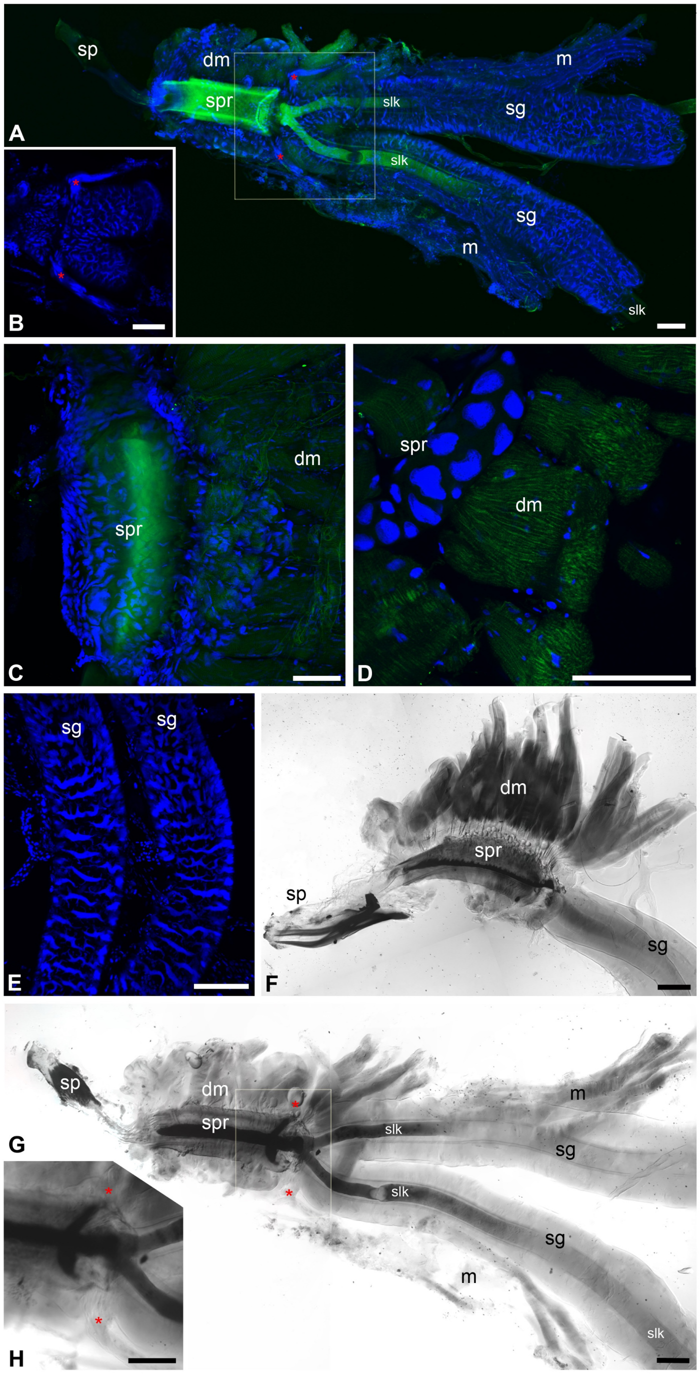

S2 Fig. The silk spinning apparatus region of cecropia. Visualization was done by double-fluorescence staining for phalloidin (green) and DAPI (blue) (A-E), and by bright-field microscopy (F-H), respectively: dorsal muscles (dm), lumen of the silk gland or silk press (lu), muscles (m), silk gland (sg), silk protein mass in the lumen of the silk glands (slk), spigot of the spinneret (sp), silk press (spr). Scale bars = 100 µm. (A), and (G) Dorsal view of the salivary glands, silk press, and surrounding muscles. (B), and (H) Cut-out of (A), and (G), respectively, depicting the outflows of salivary gland forming muscle insertions (red asterisks). (C), (D), and (F) Detailed sagittal views of the silk press and associated dorsal muscles. (E) Polyploid nuclei of the silk glands.
