# Supplementary material for: A re-evaluation of silk measurement by the cecropia caterpillar (Hyalophora cecropia) during cocoon construction reveals use of a silk odometer that is temporally regulated
Source: PLoS One. 2020 Feb 19;15(2):e0228453. doi: 10.1371/journal.pone.0228453 (PMC7029867; doi:10.1371/journal.pone.0228453)
Supplement: S3 Fig — (PDF) [file pone.0228453.s005.pdf]

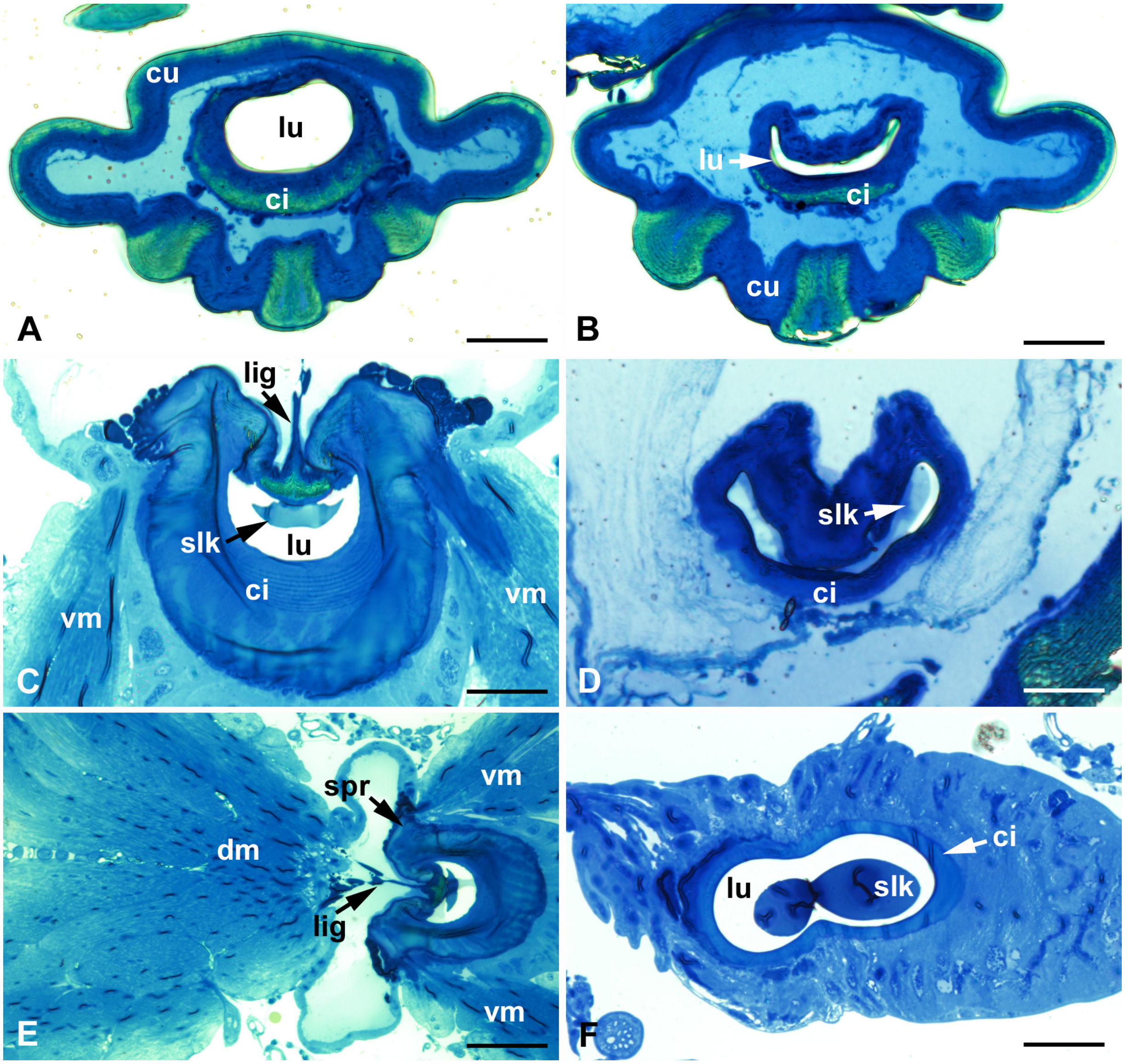

S3 Fig. Transverse sections of the cecropia spinning apparatus (light microscopy images): cuticular intima (ci), outer cuticle (cu), dorsal muscles of the silk press (dm), attaching ligament (lig), lumen (lu), silk (slk), silk press (spr), ventral muscles of the silk press (vm). (A) Cross section of the distal region of spigot. Scale bar = 50 μm. (B) Transverse section of the proximal region of the spigot showing the U-shaped lumen. Scale bar = 50 μm. (C) Transverse section of the silk press in the opened position. Scale bar = 50 μm. (D) Transverse section of the silk press in the closed position. Scale bar = 50 μm. (E) Lower magnification of (C) showing the silk press dorsal muscles with the ligament attached to the dorsal cuticle of the silk press. Scale bar = 100 μm. (F) Posterior part of the silk press at the point where the two silk glands merge. Scale bar = 50 μm.
