# Supplementary material for: Correction: A re-evaluation of silk measurement by the cecropia caterpillar (Hyalophora cecropia) during cocoon construction reveals use of a silk odometer that is temporally regulated
Source: PLoS One. 2020 Mar 12;15(3):e0230597. doi: 10.1371/journal.pone.0230597 (PMC7067433; doi:10.1371/journal.pone.0230597)
Supplement: S2 File — (PDF) [file pone.0230597.s002.pdf]

PLOS ONE

RESEARCH ARTICLE

A re-evaluation of silk measurement by the cecropia caterpillar (*Hyalophora cecropia*) during cocoon construction reveals use of a silk odometer that is temporally regulated

Hana Sehadova[1,2], **Patrick A. Guerra**[3]*, **Ivo Sauman**[1,2]*, **Steven M. Reppert**[4]*

1 Biology Centre of the Czech Academy of Sciences, Institute of Entomology, Branisovska, Ceske Budejovice, Czech Republic, **2** Faculty of Science, University of South Bohemia, Branisovska, Ceske Budejovice, Czech Republic, **3** Department of Biological Sciences, University of Cincinnati, Cincinnati, OH, United States of America, **4** Department of Neurobiology, University of Massachusetts Medical School, Worcester, MA, United States of America

* guerrapk@ucmail.uc.edu (PAG); sauman@entu.cas.cz (IS); steven.reppert@umassmed.edu (SMR)

Check for updates

OPEN ACCESS

Citation: Sehadova H, Guerra PA, Sauman I, Reppert SM (2020) A re-evaluation of silk measurement by the cecropia caterpillar (*Hyalophora cecropia*) during cocoon construction reveals use of a silk odometer that is temporally regulated. PLoS ONE 15(2): e0228453. https://doi.org/10.1371/journal.pone.0228453

Editor: Michel Renou, INRA-UPMC, FRANCE

Received: September 10, 2019

Accepted: January 15, 2020

Published: February 19, 2020

Copyright: © 2020 Sehadova et al. This is an open access article distributed under the terms of the Creative Commons Attribution License, which permits unrestricted use, distribution, and reproduction in any medium, provided the original author and source are credited.

Data Availability Statement: All relevant data are within the paper and its Supporting Information files.

Funding: This work was supported in part by the Higgins Foundation to SMR, Czech Science Foundation Grant 17-01003S to IS, and the University of Cincinnati to PAG. The funders had no role in study design, data collection and analysis, decision to publish, or preparation of the manuscript.

Abstract

The late 5th instar caterpillar of the cecropia silk moth (*Hyalophora cecropia*) spins a silken cocoon with a distinct, multilayered architecture. The cocoon construction program, first described by the seminal work of Van der Kloot and Williams, consists of a highly ordered sequence of events. We perform behavioral experiments to re-evaluate the original cecropia work, which hypothesized that the length of silk that passes through the spinneret controls the orderly execution of each of the discrete events of cocoon spinning. We confirm and extend by three-dimensional scanning and quantitative measurements of silk weights that if cocoon construction is interrupted, upon re-spinning, the caterpillar continues the cocoon program from where it left off. We also confirm and extend by quantitative measurements of silk weights that cecropia caterpillars will not bypass any of the sections of the cocoon during the construction process, even if presented with a pre-spun section of a cocoon spun by another caterpillar. Blocking silk output inhibits caterpillars from performing normal spinning behaviors used for cocoon construction. Surprisingly, unblocking silk output 24-hr later did not restart the cocoon construction program, suggesting the involvement of a temporally-defined interval timer. We confirm with surgical reductions of the silk glands that it is the length of silk itself that matters, rather than the total amount of silk extracted by individuals. We used scanning electron microscopy to directly show that either mono- or dual-filament silk (i.e., equal silk lengths but which vary in their total amount of silk extracted) can be used to construct equivalent cocoons of normal size and that contain the relevant layers. We propose that our findings, taken together with the results of prior studies, strongly support the hypothesis that the caterpillar uses a silk "odometer" to measure the length of silk extracted during cocoon construction but does so in a temporally regulated manner. We further postulate that our examination of the anatomy of the silk spinning apparatus and ablating spinneret sensory output provides evidence that silk length measurement occurs upstream of output from the spinneret.

Competing interests: The authors have declared that no competing interests exist.

Introduction

Many animals have evolved with the ability to measure quantities. The ability to perform such measurements is a key aspect of motor programs that guide ecologically relevant movement behavior in time and space [1]. For instance, several arthropods such as desert ants [2,3], salticid spiders [4], and fiddler crabs [5] appear to possess odometer mechanisms that allow them to measure distance to perform accurate and precise homing behavior across a variety of landscapes [1]. In addition to measuring distance, i.e., a measurement of how far apart two objects or points are, such a biological odometer mechanism could also function to measure the length of a three-dimensional (3D) object within one dimension, such as the length of self-generated materials like the silk fiber used for the construction of moth cocoons and spider webs.

Cocoon construction by the mature caterpillar of the cecropia silk moth *Hyalophora cecropia* (Lepidoptera; Saturniidae), represents an example of such an intriguing situation in which individuals might measure silk length culminating with the building of a cocoon with distinct, multilayered architectural features [6–10]. Constructed by late 5th instar larvae during the summer, cocoons serve as important overwintering housing for individuals during the pupal stage, and from which individuals will later eclose as adults in the following spring [10–12].

Silk used for cecropia cocoon construction is drawn from the spinneret, located in the head below the mandibles, via contact of the spinneret's tip with a substrate and the movement of the caterpillar head that draws out (extracts) silk. Cocoon construction in cecropia progresses through a stereotyped sequence of events, as described by the classic work of Van der Kloot and Williams [6–8]. After selecting a spinning site, individuals first assemble a silk scaffold that secures the cocoon at the spinning site. Using this scaffold as a foundation, individuals will then construct an outer envelope, the first of two discrete cocoon envelopes. Once the outer envelope is complete, individuals spin an internal silk scaffold for the construction of the inner envelope wherein pupation occurs.

In the current study, we postulate that successful cocoon construction in cecropia is mediated by the ability of caterpillars to measure the length of silk extracted during the cocoon construction program. To test this hypothesis, we confirmed and extended much of the work of Van der Kloot and Williams [6–8] by conducting a series of cocoon-spinning behavioral experiments to determine whether the caterpillar indeed measures the length of silk extracted during cocoon construction. We established by 3D scanning and quantitative measurements of silk weights that cocoon construction can be interrupted during spinning, but when allowed to re-spin, the caterpillar continues the cocoon construction program from where it left off. We confirmed and extended by quantitative measurements of silk weights that caterpillars will not bypass any of the sections of the cocoon during the construction process, even if presented with a pre-spun section of a cocoon spun by another caterpillar. By blocking silk output, we also demonstrated that those caterpillars do not perform the normal repertoire of spinning behaviors and, surprisingly, unblocking silk extraction 24 hr later did not restart the cocoon construction program. This suggests the existence of an interval timer that constrains the timing during which the cocoon spinning program can be executed. We used surgical reductions of the silk glands to confirm that it is the length of silk itself, rather than the total amount of silk extracted by individuals that matters. Using scanning electron microscopy, we directly showed that either mono- or dual-filament silk (i.e., equal silk lengths but which vary in their total amount of silk extracted) can be used to construct equivalent cocoons of normal size and that contain all relevant layers. We further postulate that examination of the anatomy of the silk spinning apparatus and ablation of spinneret sensory output provides evidence that silk length measurement occurs upstream of output from the spinneret. We believe that the

cocoon construction program of cecropia provides a unique example of a temporally-regulated biological odometer that measures the length of silk to execute the program.

Overview: Cecropia cocoon construction

To set the stage for our studies, we detail the known events involved in cecropia cocoon building. The cocoon construction program begins when the late 5th instar caterpillar ceases to feed and subsequently purges its gut. Gut purging occurs in the morning hours and its timing is controlled by a circadian clock [13,14]. To expel their intestinal contents, caterpillars hang their last two thoracic and anal prolegs off the branch and defecate, reducing larval weight by ca 30%. At the end of purging, which lasts 30 to 60 min, the larva begins wandering activity for 1 to 8 hrs [14], looking for the appropriate place to spin its cocoon.

Once a suitable location has been found, usually a branch of a tree or shrub, cocoon spinning initiates a highly ordered sequence of behaviors, which occur over 48 hrs of continuous spinning at the chosen spinning site [6,10] (Fig 1). Once the caterpillar begins to spin, it uses stretch-bend and swing-swing movements to first spin a scaffold, and figure-8 movements to attach the cocoon to the anchoring branch; all three movements are subsequently used throughout the entire cocoon spinning process (Fig 1B). These three fundamental behaviors of

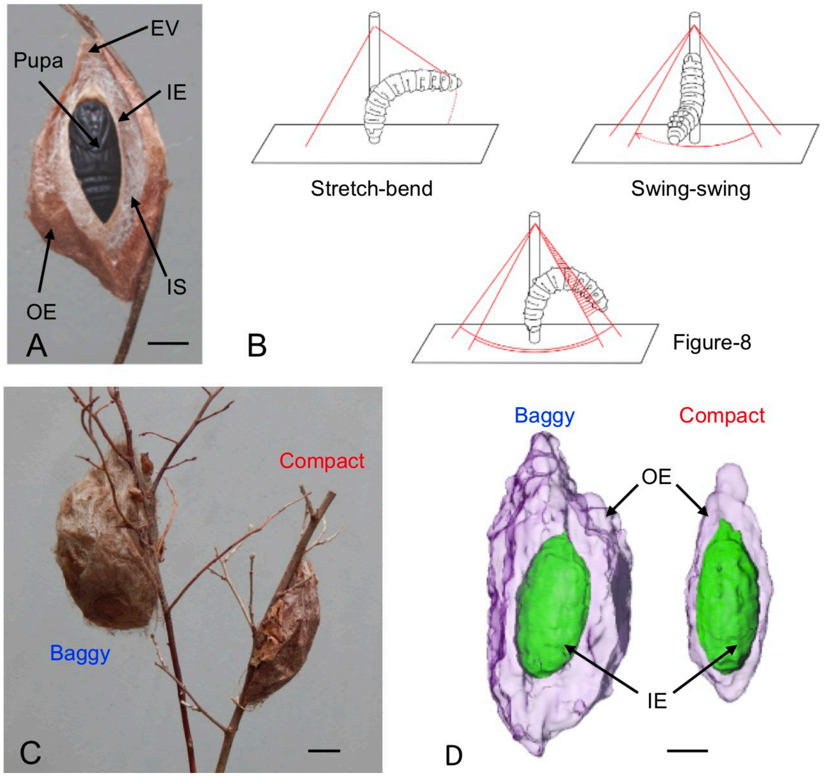

Fig 1. Cocoon construction overview. (A) Cecropia cocoon morphology. A longitudinal, sagittal section of a cocoon with encased pupa is shown with inner components labeled. (B) Spinning behaviors: Stretch-bend, attach silk via contact at one location, and using distinct pulling motions, extract silk thread and bend body to attach silk to a second location. Swing-swing, add silk to surface using back-and-forth swinging motion, while in continuous contact with the surface. Figure-8, add silk to surface using figure-8 motion, while in continuous contact with the surface. (C) Baggy and compact cocoon examples. (D) Computerized axial tomography of baggy and compact cocoons showing difference in outer envelope and intermediate space volume. Outer envelope (OE), intermediate space (IS), inner envelope (IE), escape valve (EV). Modified from Guerra & Reppert [10]. Scale bars = 1cm.

https://doi.org/10.1371/journal.pone.0228453.g001

cocoon building were initially described and named by Van der Kloot & Williams [6], by observing the cone-shaped construction of a cocoon on a vertical dowel. Guerra and Reppert [10] subsequently used a spinning arena composed of a dowel anchored to the bottom of a rectangular acrylic box to observe spinning behaviors. The boxed-environment provides a more natural context for scaffold formation (using the inner sides of the arena) and subsequent cocoon construction. Cocoons constructed in the acrylic box were identical in terms of their external shape to those found in the wild [10]. Moreover, continuous video recordings of spinning in the arena provided a real-time view of the utilization of the three behaviors in scaffold and outer envelope construction (see S1 Video).

From what appears to be an unorganized scaffold mesh emerges the beginning of the formation of the outer envelope (OE) at 8–10 hrs into spinning [10]; silk weight of the external scaffold was not measured in our studies because it is an integral part of the OE and difficult to separate. The OE is completed at 32–36 hrs of spinning. Once the OE has been fully spun, caterpillars impregnate the silk by painting the entire inner lining with an anal exudate [13]. This fluid completely coats the OE and tans it to a brownish color, which can vary greatly in hue. The OE dries and hardens, increasing OE durability. At the end of the impregnation process, caterpillars then shift to the spinning of the inner envelope (IE), anchored to the OE by the intermediate space (IS) scaffold. Complex sequences of turning behaviors help guide the cocoon construction program [7]. As part of its architecture, a cocoon possesses valves in both the outer and inner envelopes from which the fully formed adult exits the cocoon (Fig 1A). The escape valves are always oriented upward relative to the ground or the horizontal plane [6].

Across the range of habitats of the cecropia silk moth, a striking cocoon dimorphism exists in which caterpillars can spin either a large, fluffy (baggy) cocoon or a significantly smaller, tighter (compact) cocoon [9,10] (Fig 1C). There is no difference in the total weight of silk spun by the two morphs, and studies have shown that the morph difference resides in the outer envelope architecture [9,10] (Fig 1D); the baggy OE is larger and thinner than that of the compact OE, and there is more IS silk used as a scaffold for construction of the IE in baggy cocoons. There is no difference in the IE size, shape, or weight between morphs, however. Baggy cocoons appear to absorb and trap heat from infrared light and allow greater moisture permeability [10]. Cocoon dimorphism in cecropia is stochastic and may provide a bet-hedging strategy for coping with varying environmental differences that occur over its broad habitat range [10]. The vast majority of the cocoons spun in the current studies were of the compact variety. The cocoon spinning behavioral repertoire (construction behaviors and the pattern of progression for construction), the mechanisms controlling cocoon construction, and the anatomical features of the spinneret that we describe below apply to both cocoon types. We presume that all of the prior work of Van der Kloot and Williams [6–8] only examined the compact morph.

Results

A highly-ordered sequence of spinning behavior

We began our studies by showing that cocoon construction can be interrupted during spinning, but when allowed to re-spin, the caterpillars continue the cocoon construction program from where they left off, be it before or after the completion of the OE (Fig 2), as previously described [7]. In the first set of experiments, we interrupted silk deposition and the construction of the OE. Interruption was attained by removing caterpillars (n = 8) that had already spun a clearly-delineated OE encasing the caterpillar, designated an initial OE (Fig 2A, left-side of left panel); a distinct compact initial OE was formed between 15 and 21 hrs after the

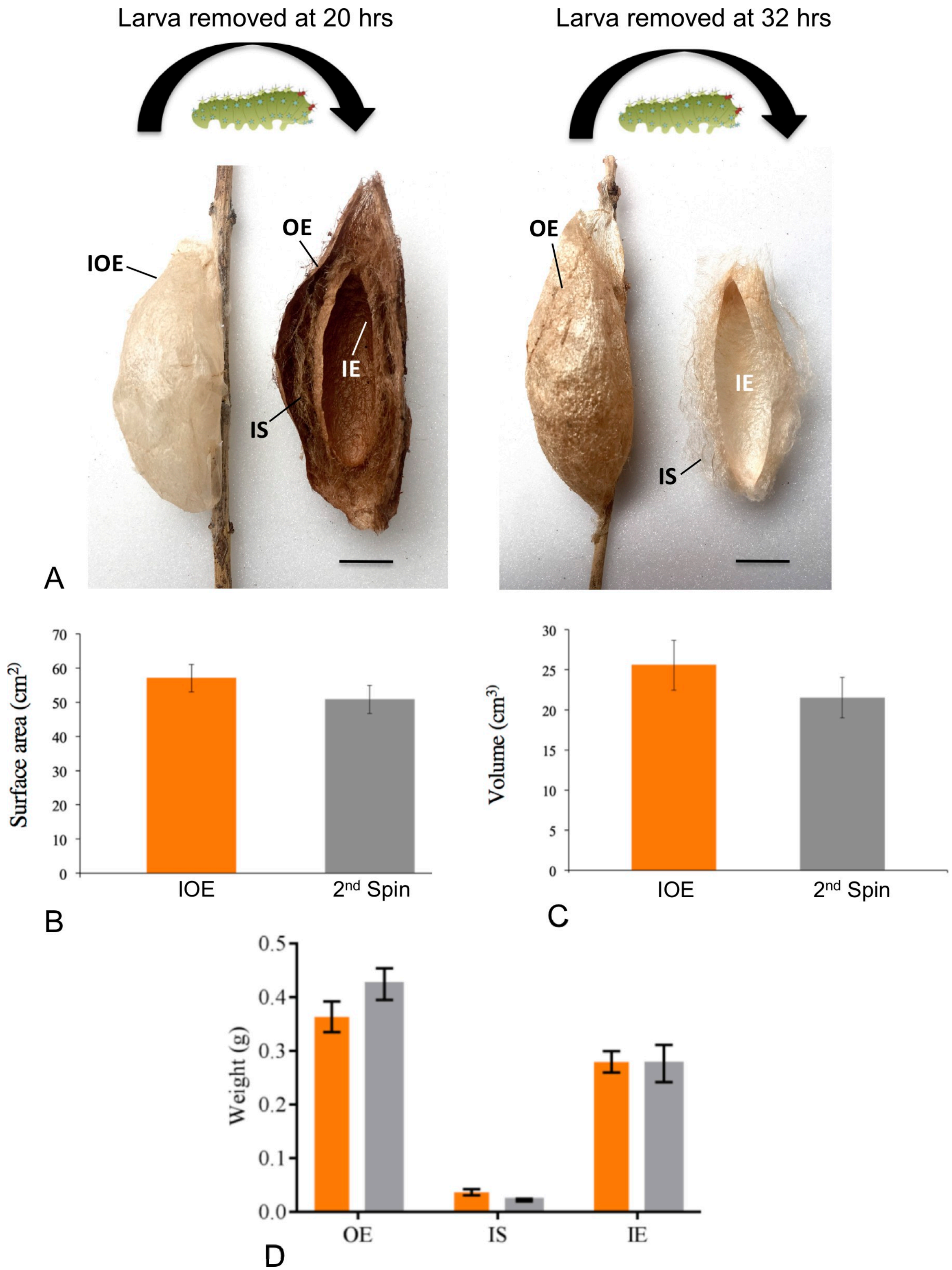

Fig 2. Interrupting silk deposition during cocoon construction. (A) Left panel. Initial outer envelope (IOE) at 20 hrs after the onset of cocoon construction (left side) and a longitudinal, sagittal section of the completed cocoon, with pupa removed, revealing all layers on 2nd spin (right side), including the outer envelope (OE), intermediate space scaffold (IS) and the inner envelope (IE). Right panel. A completed, impregnated OE at 32 hrs after the onset of cocoon construction (left side) and a longitudinal, sagittal section of the IE spun on 2nd spin (right side) with attached fragments of the IS. The shown IE weighed 0.20 gm, which was within the range of IEs collected

from field-collected cocoons. Scale bars = 1 cm. B) surface area and (C) volume comparisons between the IOE (orange) and the OE of 2nd spin cocoons (gray). (D) Comparisons between field-collected cocoons (orange) and IOE plus 2nd spin cocoons (gray) in the amount of silk for each cocoon layer (OE, IS, and IE). Values in (B), (C), and (D) are mean ± SE.

https://doi.org/10.1371/journal.pone.0228453.g002

initiation of spinning. Despite the interruption of cocoon construction, when allowed to spin again on another branch, all caterpillars continued from where they had left off by finishing the OE and proceeded to construct normal compact cocoons, in which all layers (OE, IS, IE) were present (Fig 2A, right-side of left panel). 3D scans showed that the completed OEs shared the same architectural features as those of the initial OEs, as both had similar surface areas (Paired t-test: $t_{(8)} = 1.648$, P = 0.1379; Fig 2B) and volumes (Paired t-test: $t_{(8)} = 1.404$ =, P = 0.1979; Fig 2C). We additionally found no difference in the weight of each layer (OE, IS and IE) of completed compact cocoons (initial OE plus 2nd spin, n = 3) when compared to each layer of field-collected compact cocoons (n = 10) (unpaired t-tests: OE comparison–$t_{(11)}$ = 1.0983, P = 0.2955; IS comparison–$t_{(11)}$ = 1.3452, P = 0.2056; IE comparison–$t_{(11)}$ = 0.0807, P = 0.9371; Fig 2D), nor in the total weights of completed cocoons (initial OE plus 2nd spin) when compared to field-collected cocoons (unpaired t-test: $t_{(11)}$ = 0.5438, P = 0.5974); all specimens were dried before weighing thereby minimizing any contribution of OE impregnation material (a solution containing salt crystals) to silk weights [13]. Initial OE silk represented ~22% of the total OE silk spun (21.66 ± 3.79%, n = 3). Despite constructing an initial OE with normal architectural features, on their second spin, caterpillars continued to spin an OE that is similar, but impregnated, and their combined weights suggest that a specific amount of silk is allocated for the complete construction of the OE (~60% of total silk expended for complete cocoon construction).

Interestingly, one clearly-delineated baggy OE, not included in the above data set or examined previously, was initially spun by a caterpillar and encased this individual. After removal from this initial baggy OE and on its 2nd spin, the caterpillar spun a more baggy-like complete cocoon. This very limited analysis suggested a new finding: caterpillars produce a completed OE that is of the same cocoon-morph and which shares the same architectural features as that of the initial OE (S1 Fig).

In a second experiment, we removed caterpillars after completion of the OE, signaled by its impregnation (n = 4), which occurred at 32–36 hrs of spinning. When the caterpillars were allowed to re-spin in an arena, they each spun only the IE (Fig 2A, right panel) anchored to the arena wall by an IS scaffold. These results confirmed the finding [7] that when caterpillars have spun a completed OE prior to removal, they subsequently progress to the next phase and construct an IE. We also confirmed that all three spinning behaviors are used for IE construction [7]. IE construction occurs even when caterpillars do not have access to the OE that they had previously spun. In fact, prior studies [7] show that if caterpillars begin spinning in a two-dimensional environment, in which there are no points for silk attachment in the third dimension, they spin a flat sheet of silk using only figure-8 movements. If, however, after 60% of estimated total silk has been spun as a sheet, the caterpillar is placed back into a 3D environment, the caterpillar will continue as if it had already spun the OE and will now spin an IE, by utilizing stretch-bend and swing-swing spinning movements.

Exposure to pre-spun cocoon envelopes

Following the protocol of Van der Kloot & Williams [7], we found that caterpillars will not bypass any of the two envelopes (OE, IE) of the cocoon during the construction process, even if presented with a pre-spun envelope of a cocoon spun by another caterpillar (Fig 3). For example, whether placed within a pre-spun baggy OE (Fig 3A) or compact OE (Fig 3B),

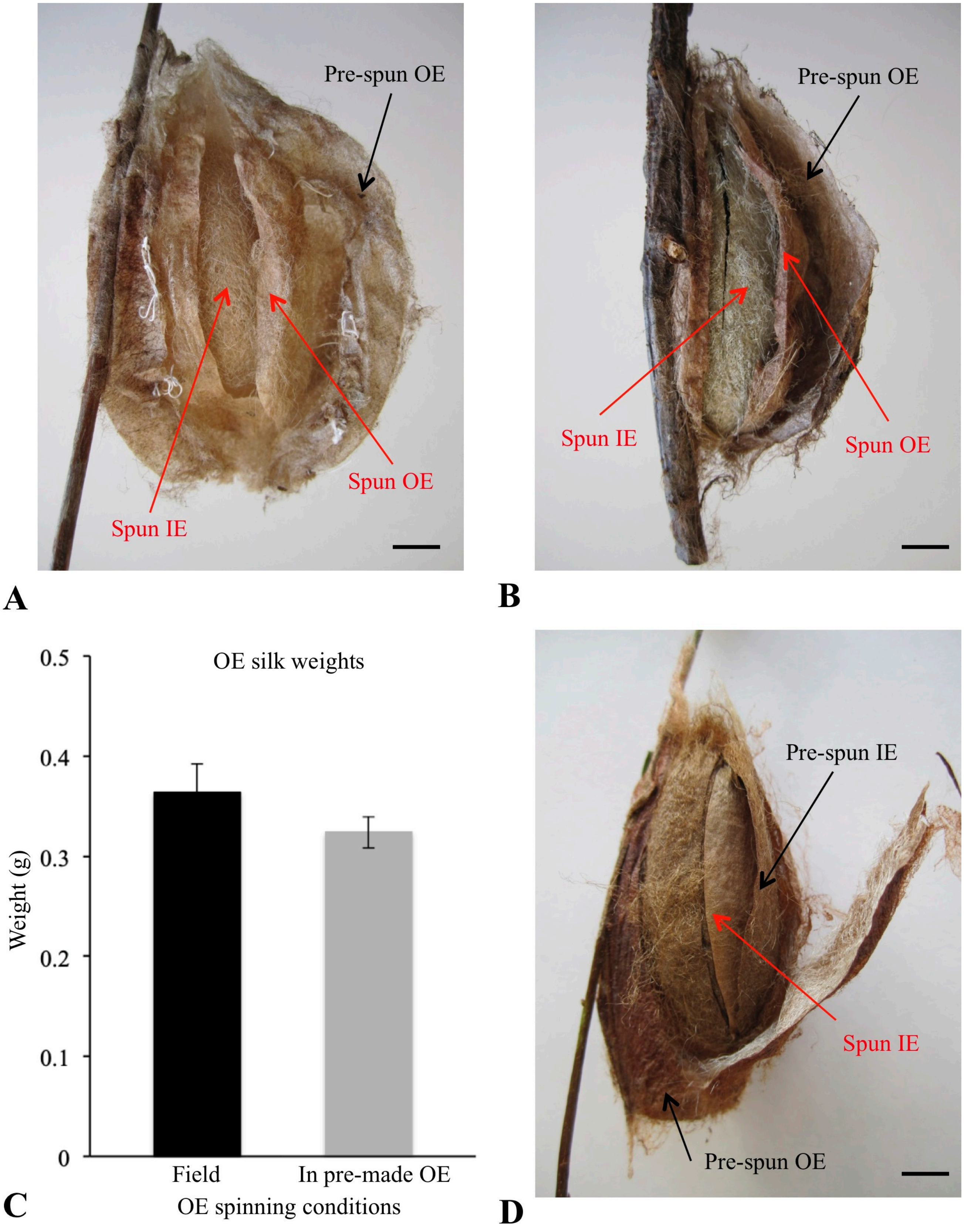
Pre-spun OE
Spun IE
Spun OE
A
Pre-spun OE
Spun IE
Spun OE
B
OE silk weights
0.5
0.4
0.3
0.2
0.1
0
Weight (g)
Field
In pre-made OE
OE spinning conditions
C
Pre-spun IE
Spun IE
Pre-spun OE
D

Fig 3. Cocoons spun by caterpillars when positioned within a pre-spun section of a cocoon. Examples of cocoons spun by caterpillars when placed inside either a pre-spun (A) baggy outer envelope (OE) or (B) compact OE. (C) Comparison of the amount of silk contained within the outer envelope of field-collected cocoons (n = 10) and that of cocoons spun in a pre-spun OE (n = 6). For both of these groups, the OE had compact outer envelope morphology. Black bar denotes field-collected cocoons and grey bar denotes cocoons that were spun in a pre-spun OE. Values are mean ± SE. (D) Example of a cocoon spun in which a caterpillar was allowed to spin its own impregnated OE prior to being placed in a pre-spun inner envelope (IE) and then allowed to continue. Scale bars = 1 cm.

https://doi.org/10.1371/journal.pone.0228453.g003

caterpillars will still construct the appropriate cocoon envelope dictated by the cocoon construction program. In these trials, all caterpillars (3 in each group, n = 6) spun a compact cocoon, even when positioned in a pre-spun baggy OE, a situation that had not been previously examined. These compact cocoons were similar to field-collected cocoons, even though they were constructed within a pre-made OE, since we found no difference in the amount of silk between the OEs of these two groups (Unpaired t-test: $t_{(14)} = 1.02$, $P = 0.33$; Fig 3C). The initial silk scaffold used for OE construction under normal conditions was missing likely due to space constrains in a pre-spun OE, making it such that the construction of a silk scaffold for attaching the OE to the 3D components of the spinning site unnecessary, due to the topography of this constrained spinning environment. Caterpillars encased in a pre-spun IE after constructing their own impregnated OE, also constructed their own IE (n = 3; Fig 3D). In this instance, an IS was also missing likely due to space constraints in a pre-spun IE, making the spinning of a scaffold also unnecessary within this constrained spinning environment. Taken together, these results confirm that caterpillars in a 3D environment must extract the silk associated with a specific cocoon envelope (OE, IE) and complete that envelope prior to moving on to the next phase of construction [7].

Description of the cecropia spinning apparatus

For normal spinning behavior, the silk glands are necessary for silk production and the proximal spinneret (silk press and spigot, see below) are the focal point of silk extraction. We thus examined the anatomy of the entire spinning apparatus and any associated sensory structures that could connect silk extraction activity with the brain, the known control site for cocoon construction [8]. The anatomical layout of the spinneret and associated silk glands was also important for performing subsequent spinneret blockade and silk gland excision experiments, and their interpretation.

In the late 5th instar caterpillar of the cecropia moth, contralateral tubular silk glands represent most of the caterpillar body mass. The glands run alongside the gut on either lateral side of the body and are separated into a posterior, middle, and anterior region with respect to their position and morphological structure (Figs 4 and 5; S2 and S3 Figs); for schematic see Fig 6A. The middle part has a more or less uniform thickness throughout the entire length and a distinct loop in the dorso-ventral orientation is created in each body segment, while the anterior and the short, most posterior parts are notably narrower and almost straight. Each silk gland is lined with cells containing characteristic polyploid nuclei and an inner lumen containing a mass of secreted silk proteins. A thin layer of cuticle called the cuticular intima separates these two parts of the silk gland. The very thin anterior region is characterized by the fusion of paired silk glands into a single common duct inside the head capsule. The common duct then enters anteriorly to the structure referred in Lepidoptera as the silk press that continues by a tubular cone-shaped structure called the spigot of the spinneret. Importantly, silk press diameter can be regulated by its surrounding musculature (for details of the silk press musculature see S1 Text). The spigot ultimately projects outside of the head capsule from which silk is extracted. Two sensilla of the spinneret, potential sensory monitoring structures for cocoon construction (see below), are located ventro-medially from the labial palps just below the base

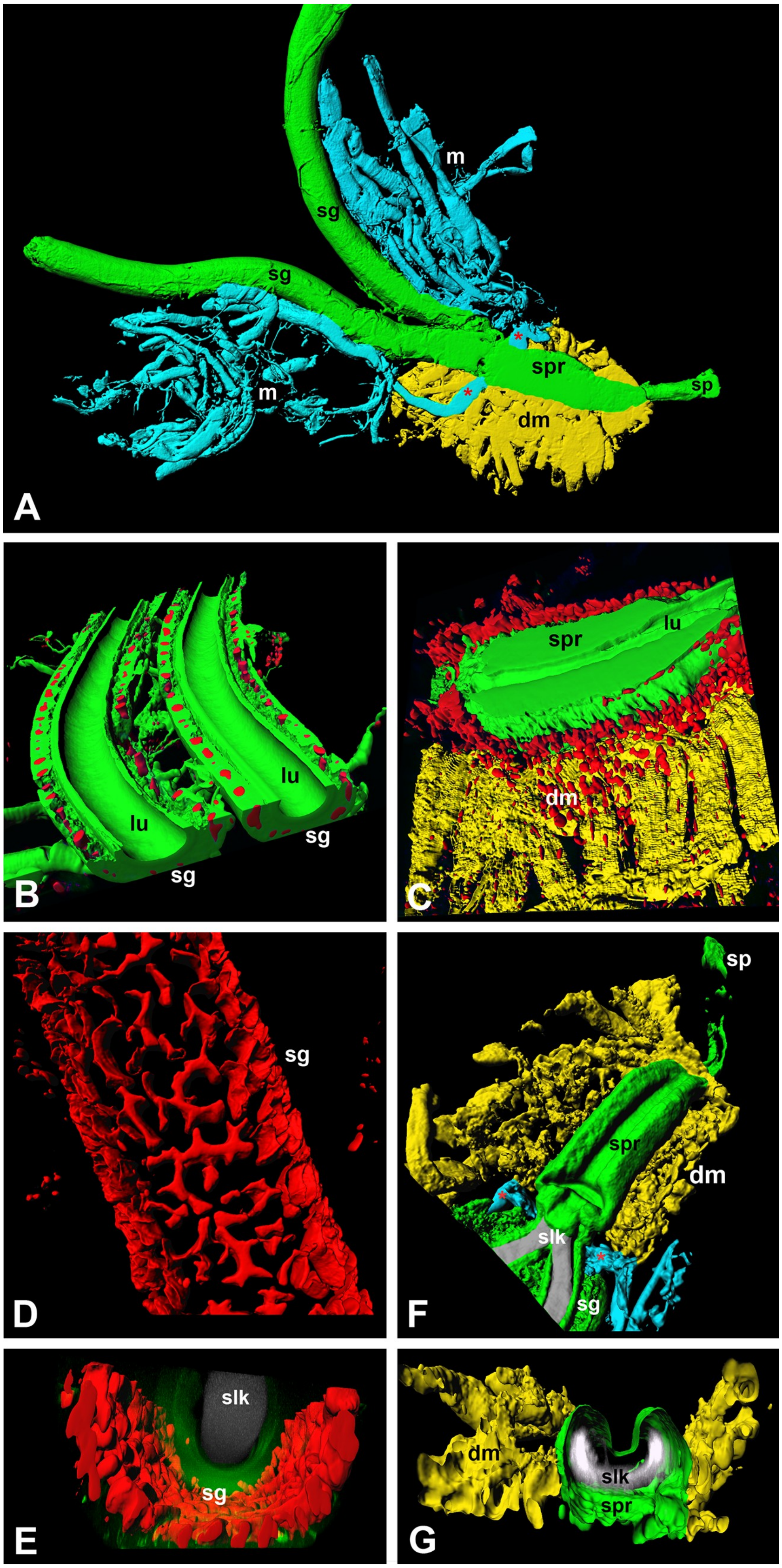

m
sg
sg
spr
sp
m
dm
A
lu
lu
sg
sg
B
spr
lu
dm
C
sg
D
sp
spr
dm
slk
sg
F
slk
sg
E
dm
slk
spr
G

Fig 4. Morphology of the late 5th instar caterpillar spinning apparatus and surrounding tissues. 3D perspective views were obtained by volume rendering of multiple confocal stacks stained by phalloidin (artificially colored–green, blue, yellow) and the nuclear stain, DAPI (colored in red). (A) Ventral view of the anatomical organization of the silk spinning apparatus. (B) Longitudinal section through the salivary glands. (C) Sagittal section through the silk press and the dorsal silk press muscles. (D), and (E) 3D reconstruction of the polyploid nuclei of the silk glands in longitudinal, and lateral view, respectively. (F), and (G) Dorsal view and vertical section, respectively, of the silk press and surrounding dorsal muscles. Abbreviations: dorsal muscles of the silk press (dm), lumen of the silk gland or silk press (lu), silk gland (sg), silk protein mass in the lumen of the silk glands (slk), spigot of the spinneret (sp), silk press (spr), ventral muscles of the silk press (vm). Outflow of the salivary gland creating a muscle insertion (red asterisk).

https://doi.org/10.1371/journal.pone.0228453.g004

of the spigot in a striated region of cuticle (Fig 5F and 5G). A comparison of spinning apparatus anatomy among moth species is provided in S2 Text. One differentiating feature that stands out is that the silk glands of saturniid moths like cecropia are more elaborate compared to those of the non-saturniid moths examined.

Blocking silk extraction through the spinneret

We found that preventing caterpillars from extracting silk from the spinneret spigot in a normal manner, as cocoon construction was initiated, compromised the cocoon construction program (behavior and the construction of the actual cocoon) and pupation timing (Fig 6). Control caterpillars that had their spinnerets blocked with super glue for one hr (n = 3) extracted silk normally and constructed normal cocoons (each with the normal three layers; OE, IS, IE) using the three basic spinning maneuvers, once their blocked spigot was severed (Fig 6B). Treatment caterpillars (n = 4) that had their spinneret blocked with super glue for 24 hrs, on the other hand, did not produce any silk and all four treatment caterpillars only performed figure-8 head movements and wandering-type locomotion (Fig 6C); the figure-8 behavior has been described as primitive and similar to the side-to-side searching behavior used by feeding caterpillars [7]. The 24-hr blocked caterpillars did not display the complete suite of behaviors that are typical of caterpillars during the cocoon construction process (e.g., addition of stretch-bend, swing-swing movements), as found in the controls.

At 24 hrs, we attempted to restart silk extraction and rescue the normal repertoire of spinning behaviors by severing the blocked spigot (Fig 6B). However, we found that there was still no silk extraction and that those caterpillars continued to only perform figure-8 head movements and wandering-type locomotion. In total, the treatment caterpillars performed only figure-8 head movements and wandering-type locomotion for a total period of 12.5 ± 1.19 days (n = 4) prior to the start of pupation. During the time that treated caterpillars were still engaging in wandering behavior, all three control caterpillars pupated normally in their cocoons.

These results show that without silk extraction through the spinneret, the normal cocoon spinning behaviors are not manifested. Furthermore, if the silk spinning program is blocked/delayed for 24 hrs, the time during which under normal circumstances most of the OE would have been completed (i.e., ~50% of the cocoon would have been constructed), silk can no longer be extracted from an open spigot, and the cocoon construction program is completely disrupted and pupation is delayed. These results are consistent with the involvement of a temporally-regulated silk odometer, in the cocoon construction program. The interval timer aspect starts with the beginning of silk extraction after wandering, and it stops 48 hrs later with the completion of cocoon construction. During this interval, the actual passage of silk through the spinneret controls cocoon construction, the normal expression of spinning behaviors during construction, and the natural developmental progression of individuals from the larval to the pupal stage.

Notably, there appears to be a critical time between 1 and 24 hrs, during which the blockage of the spinneret irreversibly inhibits silk extraction and the expression of the normal spinning

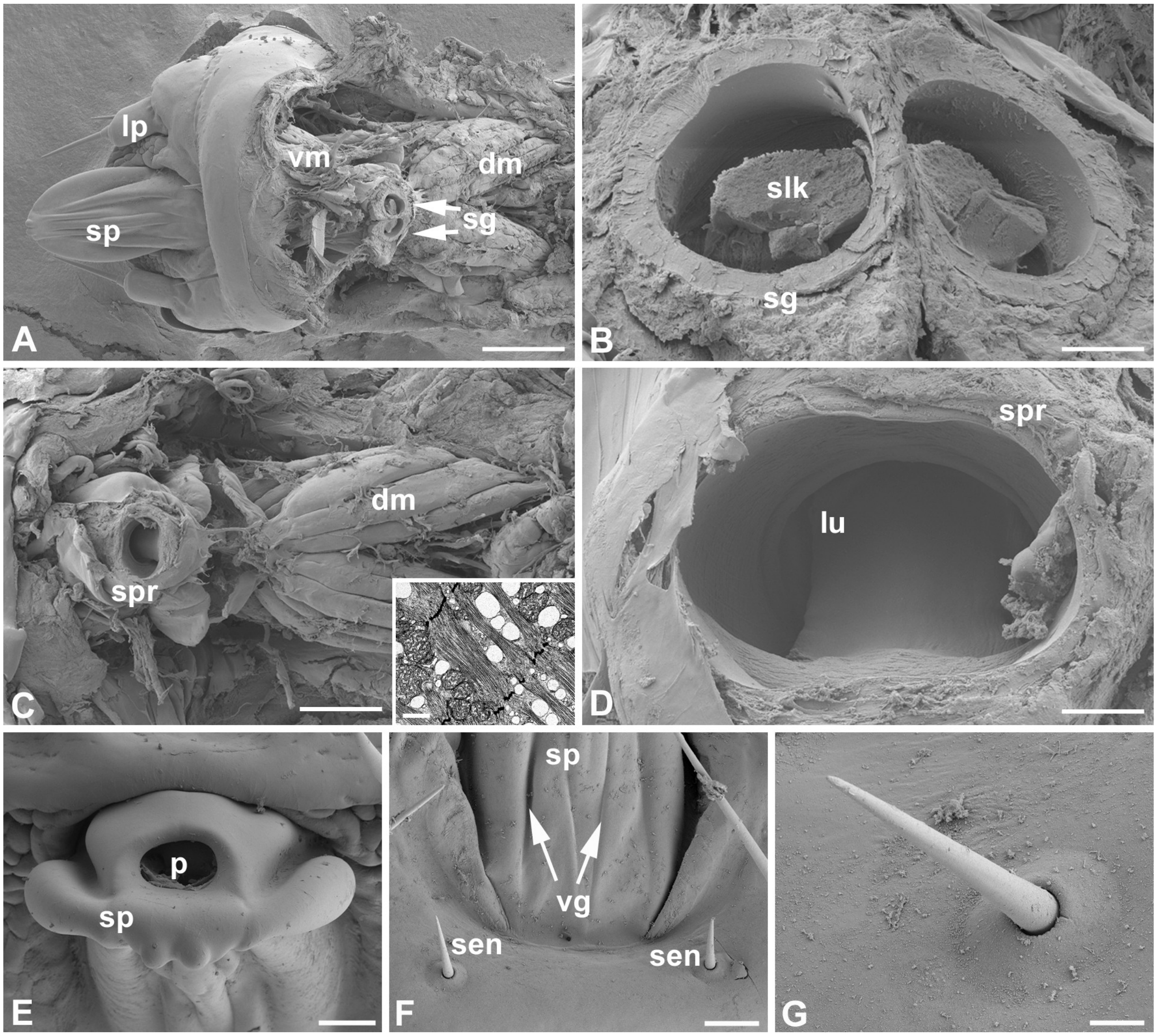

Fig 5. The cecropia cocoon spinning apparatus and sensilla. Scanning electron micrographs of the partially dissected last larval instar spinneret and surrounding tissues: (A) Ventral view depicts the anterior parts of the silk glands just before their fusion into the posterior part of the silk press (arrows); dorsal and ventral muscles of the silk press are also clearly visible. Scale bar = 200 μm. (B) Detailed view showing the silk protein mass in the lumen of the anterior part of the silk glands. Scale bar = 20 μm. (C) Ventral view of the posterior part of silk press with the exposed dorsal muscles. Scale bar, 200 μm. Inset: Transmission electron micrograph of the silk press dorsal muscle longitudinal section. Scale bar = 1 μm. (D) Lumen of the posterior part of the silk press. Scale bar = 20 μm. (E) Detail of the frontal view demonstrating the architecture of the cecropia spigot. Scale bar = 30 μm. (F) Ventral view of the spigot depicting the spigot ventral grooves (arrows), and the location of the spinneret sensilla. Scale bar = 40 μm. (G) Detail of the spinneret sensillum. Scale bar = 10 μm. Abbreviations: Dorsal muscles of the silk press (dm), labial palp (lp), lumen of the posterior part of the silk press (lu), pore of the spigot (p), spigot sensilla (sen), silk glands (sg), silk protein mass in the lumen of the silk glands (slk), spigot of the spinneret (sp), silk press (spr), ventral grooves of the spigot (vg), ventral muscles of the silk press (vm).

https://doi.org/10.1371/journal.pone.0228453.g005

behaviors. Importantly, severing the anterior part of the spigot itself does not alter cocoon spinning, as evident in the control animals, when the spigot was severed at 1 hr after blockage–all spun normal cocoons.

The length of silk passing through the spinneret dictates spinning progress

As previously performed [7], we examined cocoon construction after severing various lengths of the silk glands to restrict the quantity and quality of silk exiting the spinneret (Fig 7). We

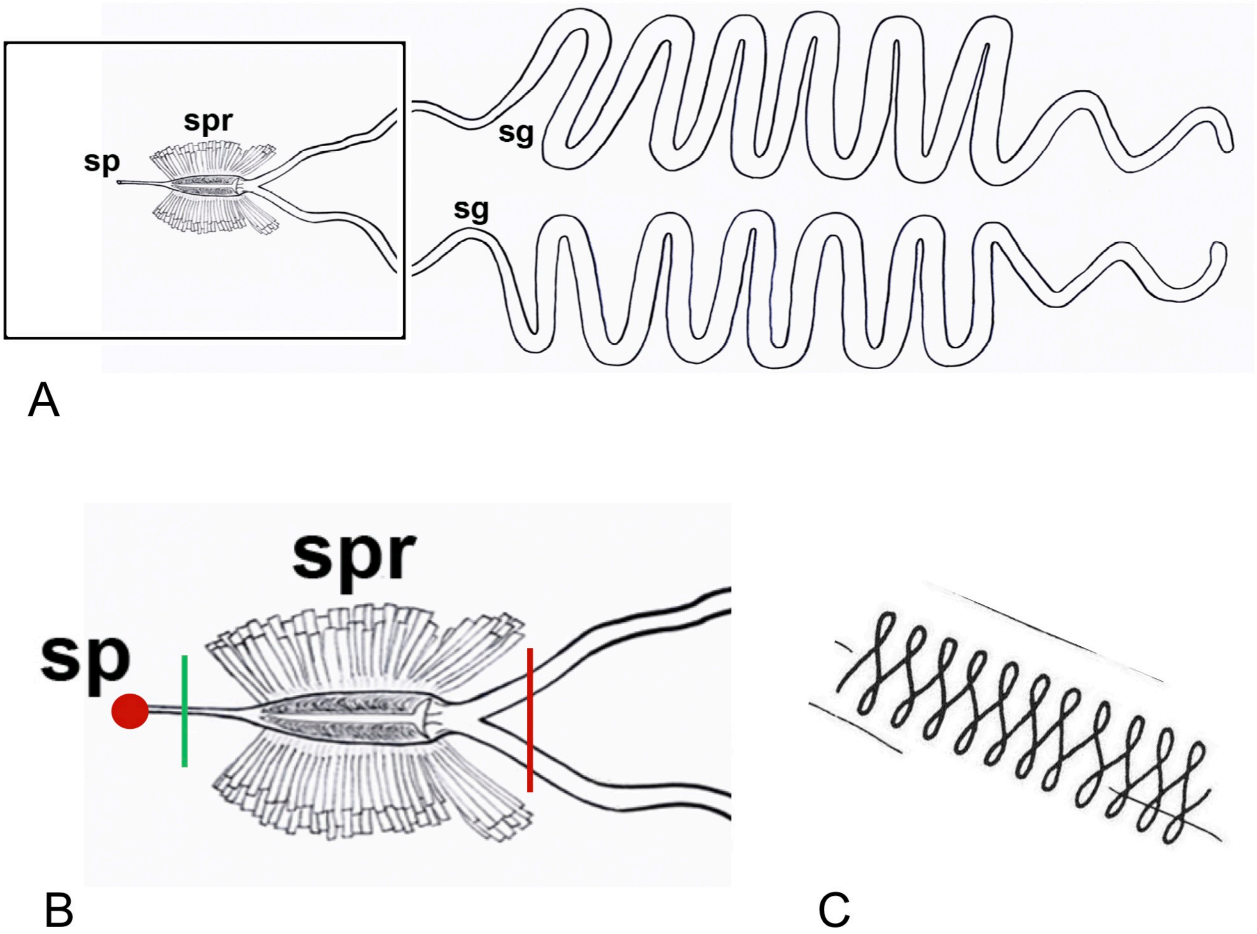

Fig 6. Spinneret blockage. (A) Cartoon of spinning apparatus of the cecropia caterpillar showing the posterior-positioned paired silk glands (sg) and the anterior-positioned spinneret components (boxed) including the silk press (spr) and outlet spigot (sp). (B) Enlarged view of the spinneret showing regions of silk blockage (red circle), severed spigot (green vertical line), and severing (or removal) of both silk glands (red vertical line). (C) Figure-8 trail of wandering caterpillars with blocked spigot. From Scarbrough et al., 1977.

https://doi.org/10.1371/journal.pone.0228453.g006

did not, however, remove both silk glands at spinning onset, which had been previously shown to disrupt spinning behaviors (Fig 6B), as only figure-8 movements were exhibited for the 5–6 days prior to pupation [7]. This was similar to what we found when the spinneret was blocked by superglue for 24 hrs, but different in that spinneret blockage also markedly disrupted the timing of pupation.

When we severed one silk gland at its anterior end, the resulting compact cocoon structure was virtually identical to intact control cocoons in terms of size and the cocoon layers, that is, it contained the OE, IS, and IE (Fig 7A and 7B). However, the one silk gland-severed cocoons weighed 50% less than unoperated controls (Fig 7C). Given that silk normally exits the spigot as dual silk filaments of fibroin cemented together by sericin proteins, it has been reasoned that the reduction in cocoon weight was due to the use of monofilament silk from the remaining intact silk gland for cocoon construction, rather than using the normal dual filament silk from the two glands [7].

We extended and confirmed these assumptions by using scanning electron microscopy to directly examine silk fiber content of the silk-gland severed cocoons. The results showed that the silk composition of the silk-gland severed cocoons was indeed all monofilament,

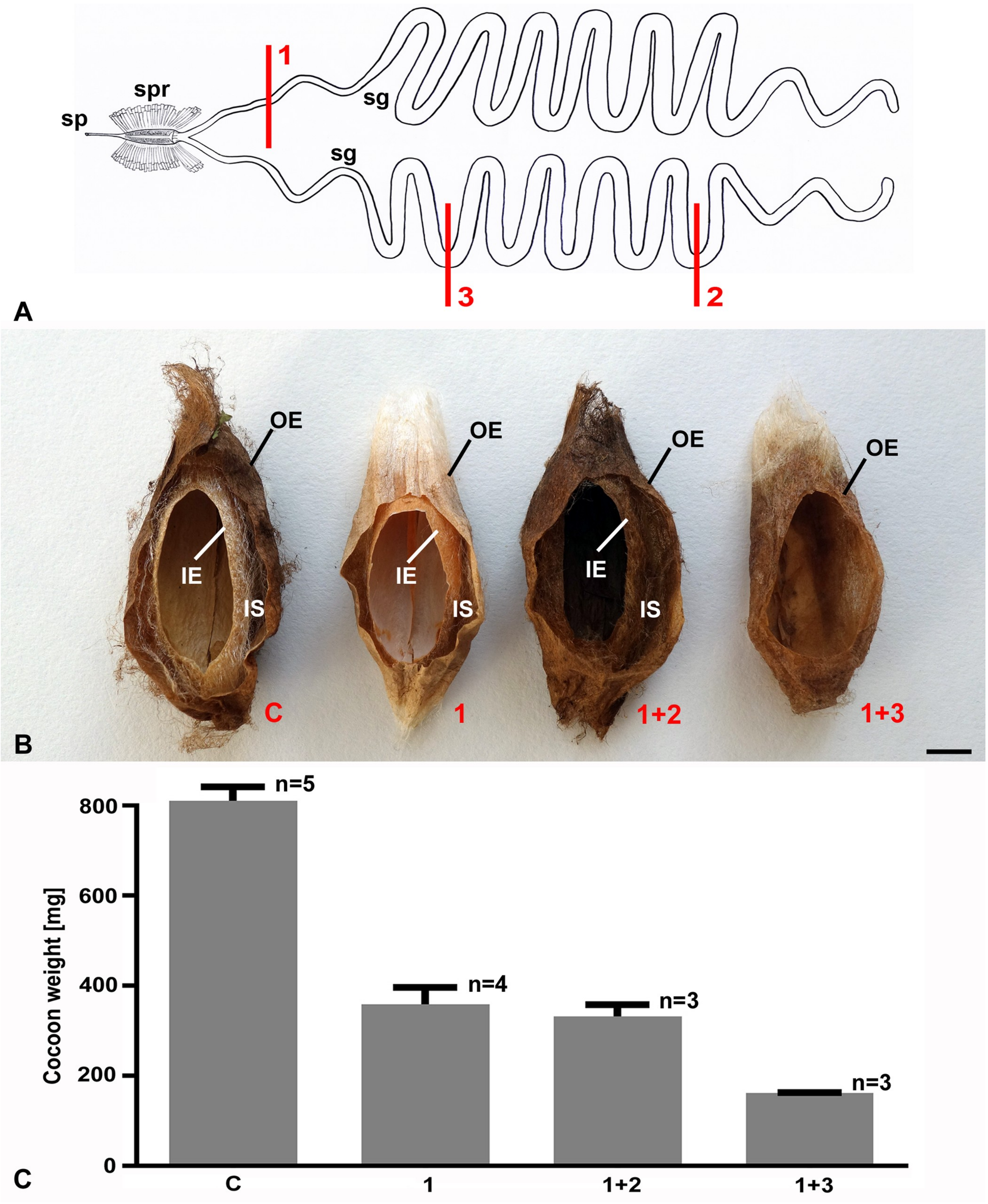

sp
spr
sg
sg
1
1
3
2
A
OE
IE
IS
C
OE
IE
IS
1
OE
IE
IS
1+2
OE
1+3
B
800
600
400
200
0
Cocoon weight [mg]
n=5
n=4
n=3
n=3
C
1
1+2
1+3
C

Fig 7. Surgical manipulations of the cecropia last larval instar silk glands and resulting cocoon structures. (A) A cartoon of the silk gland surgical ablations. Experiment #1, the entire body of one silk gland was severed, experiment #2, same as #1 plus posterior 25% of the other gland ablated, experiment #3, same as #1 plus posterior 75% of the other gland severed. Abbreviations: Silk gland (sg), spigot of the spinneret (sp), silk press (spr). (B) Longitudinal, sagittal sections of the compact cecropia cocoons spun, with pupae removed, after the silk gland surgical manipulations. From left to right, cocoon spun by a control intact larva (n = 5), surgical manipulations #1 (n = 4), #2 (n = 3), and #3 (n = 3), respectively, as described above. Note, that control larvae as well as larvae from the surgical manipulations #1 and #2 completed both the outer envelope (OE) and the inner envelope (IE), with its scaffold (IS), of the cocoon. The larvae from the experiment #3 spun only the OE. Scale bar = 10 mm. (C) Weights of the cocoons (mean ± SE) from the control and the three surgically treated groups described in (B).

https://doi.org/10.1371/journal.pone.0228453.g007

accounting for the reduction in total cocoon weight (Fig 8). When one silk gland was severed at its anterior end, and the posterior 25% of the other gland was also severed, the resulting cocoons were the same as those with only one silk gland severed (Fig 7B and 7C). However, when one silk gland was severed in combination with 75% of the other silk gland, the resulting cocoons contained only a normal sized OE–the IE with its IS was missing and this alteration in cocoon construction was reflected in a further reduction in cocoon weight (Fig 7B and 7C).

These results suggest that with silk gland reductions, the normal layers of the cocoon (OE, IS, IE) depend on the length of silk available, be it mono- or dual- filament silk fiber (see also ref 7). Moreover, our results confirm that it is the length of silk extracted through the spinneret and NOT the amount of silk left in the silk gland (see last gland reduction experiment) that regulates the normal progression of the cocoon construction program over the 48-hr period of silk extraction [7]. This suggests that silk measurement occurs downstream from the silk glands, but upstream of spinneret output (see below).

The potential role of spinneret sensilla in cocoon construction

To test whether the spinneret sensilla that are located in close vicinity to the spigot contribute to the control of cocoon construction (Fig 5F), we performed neuronal tracing from the sensilla and then examined the effect of sensilla removal.

Tracing from the spinneret sensilla. Like most of the insects' mandibular head segments [15], the spinneret sensilla are innervated from the suboesophageal ganglion (SOG). The sensory inputs from the sensilla are transmitted via two labial trunk nerves that terminate and arborize in the labial neuromere of the SOG (Fig 9). Functionally, the SOG in all insects interacts closely with the supraoesophageal ganglion (SPG, the brain). Through its local sensory processing neurons and the output neurons, the SOG relay sensory information to the higher centers in the brain for further processing [16] and, in this way, the sensilla may communicate silk extraction activity through the spinneret to the brain–a postulate we tested below.

Effects of removal of spinneret sensilla on the cocoon construction program. The potential roles of the spinneret sensilla on cocoon construction were examined by surgical extirpation, just prior to spinning. The spinneret sensilla were surgically removed as follows: 1) Removal of one spinneret sensillum (n = 4: 2x left sensillum, 2x right sensillum), and 2) removal of both spinneret sensilla (n = 5). Importantly, all operated larvae produced normal cocoons containing double-stranded silk, with all three layers (OE, IS, IE) present in each. This finding suggests that silk-length measurement resides upstream from spinneret output and may reside in the proximal spigot and/or silk press.

Discussion

Our results, along with those of Van der Kloot and Williams [6–8], provide a strong basis for proposing that a silk odometer controls the cocoon construction program of cecropia caterpillars. This odometer measures the length of extracted silk during cocoon construction, with each of the discrete events of cocoon spinning controlled by the length of silk that has passed

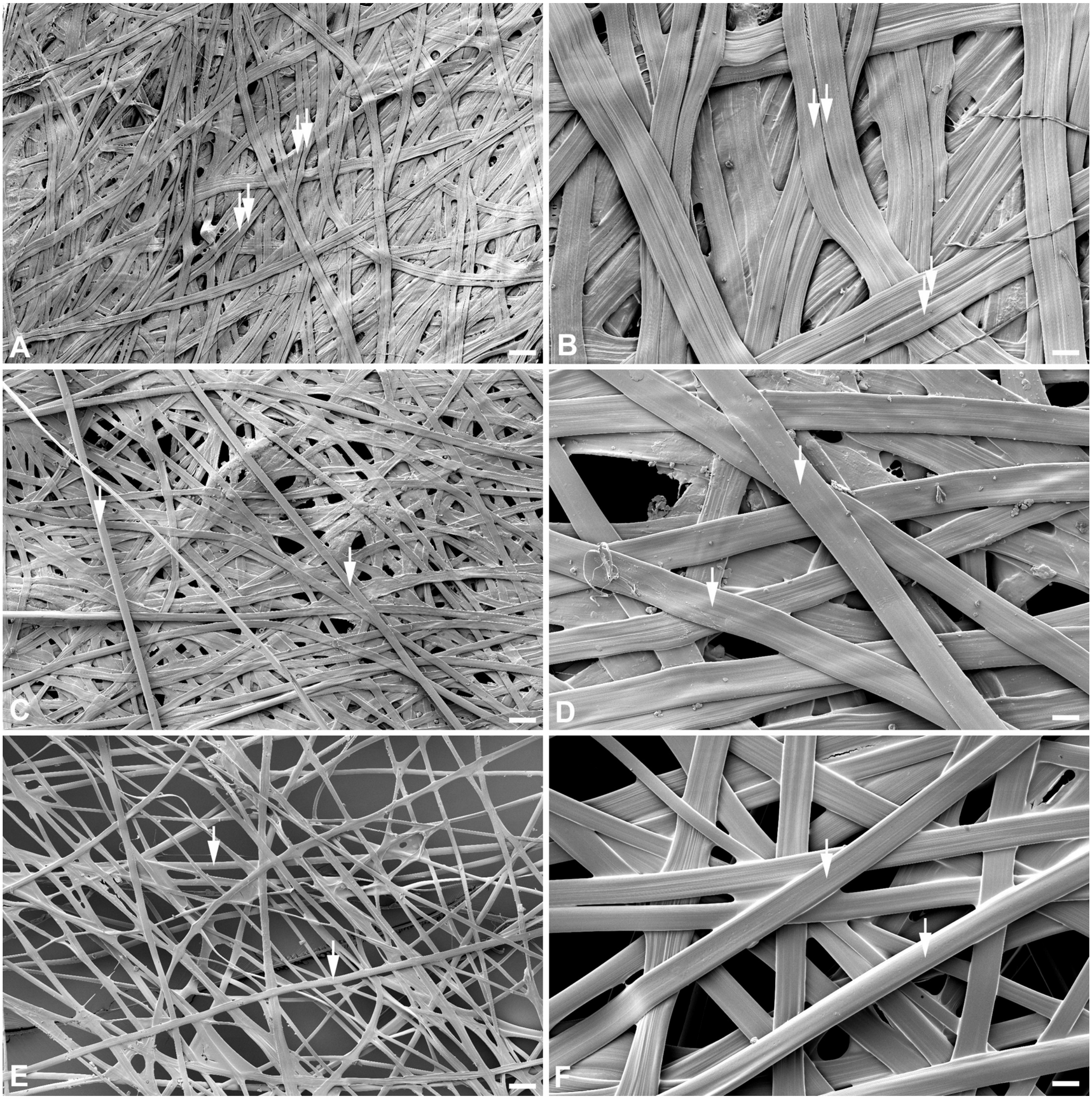

Fig 8. Scanning electron micrographs of cecropia cocoons. (A, B) A microstructure of the cocoon silk fibers spun by a control (intact) last instar larva. The double arrows indicate the dual silk filaments. (C, D) A microstructure of silk fibers spun by an operated larva with one entire silk gland surgically severed. Note the singlet silk filaments (single arrows). (E, F) The same as in (C) and (D), but 75% of the other silk gland was also surgically removed. Scale bars (A), (C), and (E) = 100 μm, (B), (D), and (F) = 22 μm.

https://doi.org/10.1371/journal.pone.0228453.g008

through the spinneret; the behavioral aspects of the odometer could be viewed as a fixed action pattern because they are stereotyped, complex, species-specific, triggered (see below) and

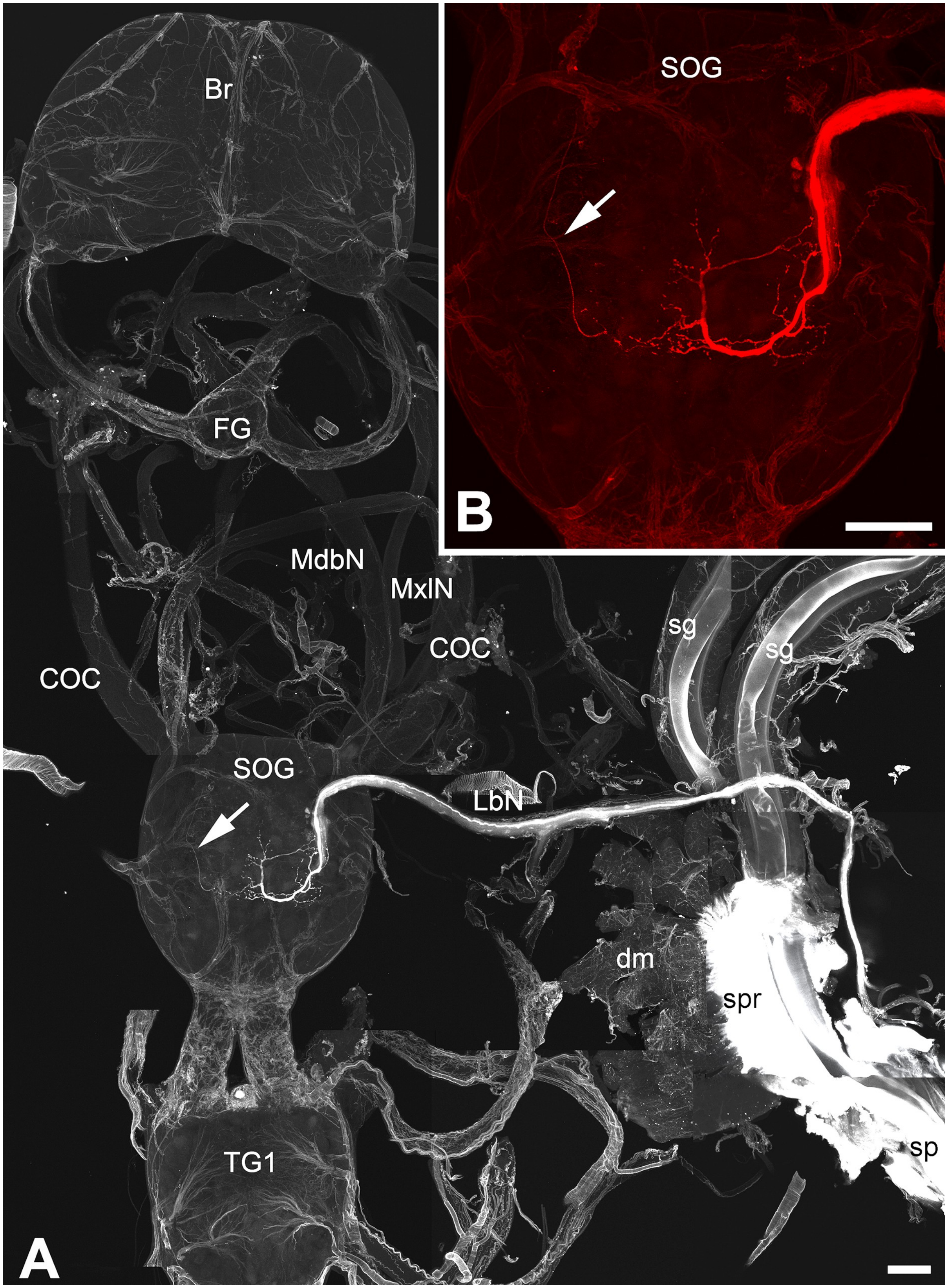

Br
FG
MdbN
MxlN
COC
COC
SOG
LbN
sg
sg
dm
spr
sp
TG1
A
SOG
B

Fig 9. Neuronal projections. Axonal projections from the spigot (spinneret) sensilla of *H. cecropia* last larval instar: (A) An overview of the whole cephalic ganglion together with the dissected spinning apparatus. (B) A detail of the suboesophageal ganglion (SOG). The arrows depict the fine extension and arrborization of the neuronal projection from the contralateral labial nerve. Scale bars = 100 μm. Abbreviations: Brain (Br), circumoesophageal connective (COC), dorsal muscles (dm), frontal ganglion (FG), labial nerve (LbN), mandibular nerve (MdbN), maxillary nerve (MxlN), silk gland (sg), suboesophageal ganglion (SOG), spigot of the spinneret (sp), silk press (spr), first thoracic ganglion (TG1).

https://doi.org/10.1371/journal.pone.0228453.g009

independent of experience [17]. The silk odometer appears to have different OE settings for construction of either the baggy or compact cocoon morphs. That the spinneret is important for the control of the length-coupled behavioral repertoire is reinforced by the finding that either mono- or dual-filament silk can be used to fully construct a cocoon containing both the OE and IE (Fig 7; ref 7).

A fascinating aspect of our work is the discovery that the measurement of silk length by the cecropia caterpillar occurs within the confines of an interval timer, as suggested by our spinneret blockage experiments. Similar studies by Van der Kloot & Williams [7] used paraffin to block silk extraction at the onset of spinning behavior. They reported that those caterpillars continue to exhibit turning movements measured in a closed-cylinder recording device. However, no direct observations of the spinning behaviors (stretch-bend, swing-swing, figure-8) were reported, and efforts to reinstate silk extraction by opening the spinneret spigot after blockage were not reported. Thus, the critical feature of our spinneret blockage experiment was to determine whether the cocoon construction program could be restarted by opening the spigot, after a sustained period (24 hrs) of blockage. It appears that the 24-hr delay was beyond the confines of interval timer activation thereby disabling the cocoon construction program. It is certainly possible that after a less extreme spinning delay, say of 12 hrs or even 18 hrs, that the interval timer could reset to start at those times triggering the fixed action pattern of spinning behavior to its completion over the subsequent 48 hrs. Further studies are needed to more precisely clarify the interval timer constraints.

The cocoon spinning program of cecropia is intimate, as all of the events are contained within the small confines of the cocoon. Moreover, the ability to measure the length of silk that specifically facilitates proper behavior (i.e., successful cocoon construction) appears, as mentioned above, to not involve any specific previous learning, as each caterpillar builds a single cocoon during its lifetime. This stands in stark contrast to the vast majority of odometer measurement programs utilized by other invertebrates, which occur over distances required for homing that rely on the sensing of external cues [1].

When we examined the potential importance of sensory input from the spinneret sensilla for cocoon construction, we were surprised that extirpation of the sensilla had no detectable influence on cecropia cocoon spinning behavior. We have also shown that the anterior tip of the spigot of the cecropia spinneret is not necessary for normal cocoon construction. Our results suggest that silk length measurement in cecropia caterpillars potentially involves an innate mechanosensory mechanism arising from the proximal spinneret. We propose that the silk press may provide such a mechanism through its muscle-regulated activity. It is also possible that there are multiple, redundant sensory pathways along the entire silk spinning apparatus (from silk glands to spinneret) involved in regulating spinning behavior that are yet to be discovered.

The cocoon building program of the cecropia caterpillar appears to have broad implications in that it likely applies to a number of other saturniid moths that also have elaborate silk gland morphology (S2 Text). In addition to cecropia, the other members of the *Hyalophora* genus spin similar multilayed cocoons with an OE, IS, and IE. However, in some species, e.g., *H. columbia columbia* and *H. columbia gloveri*, the IS is markedly reduced to undetectable [18].

Moreover, *Antheraea pernyi* builds a cocoon that also likely uses a silk measuring device, but there is no separation between the OE and IE, as the two envelopes are only differentiated by behavioral criteria [19]. A similar construction program likely extends to other members of the *Antheraea* genus, which all spin morphologically similar cocoons. Moreover, there are many other saturniid moths that spin complex, multilayered cocoons that likely utilized a cocoon construction program that depends on the measurement of silk length, similar to that of cecropia [18].

As emphasized before, the cocoon construction program of cecropia and the other saturniids discussed above is a one-time event in the life of the caterpillar. And the program appears to be less flexible and less plastic than that of other non-saturniid insects that use silk for building structures. For example, as highlighted by Lounibos [13], tent caterpillars can adjust their communal tents over time [20,21], bagworm and caddisfly larvae can adjust their case construction, e.g., they can repeat stages as necessary, depending on the context [22,23], and webspinners can repair or modify their existing silk domiciles [24]. Similarly, orb-weaving spiders can display high-levels of behavioral plasticity during the web spinning process, as individuals can repair their existing webs [25] and spin multiple webs over time with each web adapted in size and shape to the spatial constraints of the topography of the location for spinning [26]. This behavioral flexibility in web spinning in orb-weaving spiders, manifested by adjustments during web construction, appears to be neither pre-programmed nor learned [27].

To complete a discussion of all that is currently known about the silk odometer program in cecropia and to put our work in context, it is important to discuss prior studies of the location of the central control system that regulates and integrates the cocoon construction program. As previously mentioned, it has been shown that the brain is the control center for cecropia cocoon spinning, as isolating the brain from inputs leads to no spinning behavior [8]. But what specific brain structures are involved in cocoon construction? The central complex, a logical area that comes to mind for involvement in cocoon spinning behavior because of it known role as a sensory-motor integration center in adult insects [28], is less well defined in the 5th instar moth caterpillar than it is in the adult [29]. Furthermore, midline lesions in the cecropia caterpillar that would disrupt the central complex do not alter normal cocoon spinning behavior [8].

Extensive electrocautery lesions of the brain, on the other hand, have shown that the mushroom bodies (MB) are an important brain center necessary for the cocoon construction program in cecropia; unilateral MB lesions lead to the spinning of a flat pad of silk without evidence of the normal division into two layers (e.g., OE and IE) [8]. Moreover, study of metamorphic brain development shows that the mature caterpillar brain contains a well-formed MB with all the lobes present in the caterpillar that are found in the adult moth [29]. The involvement of the MBs in cocoon spinning (i.e., motor activity) is unusual as their main role appears to be in learning and memory for a variety of systems, including olfaction, associative memory, and sleep [30]. Other examples of proposed MB function in the motor activity of insects, includes courtship movements in the grasshopper [31] and locomotor activity in *Drosophila* [32]. The MBs have been thought to act as a coincidence detector, integrating multiple inputs [33,34]. Thus, the role of the MBs in cecropia cocoon spinning may be an example of the role MBs play in the multiple-input generation or coordination of the motor program. This would be consistent with their proposed role in the measurement of silk length during cocoon construction [35].

Cocoon construction in cecropia is a fascinating motor program requiring further study (Fig 10). Brain gene expression studies, calcium imaging, and multiunit electrical recording by telemetry during construction would be informative. We predict that further understanding of the neural substrates involved in cecropia cocoon construction, in particular those involved

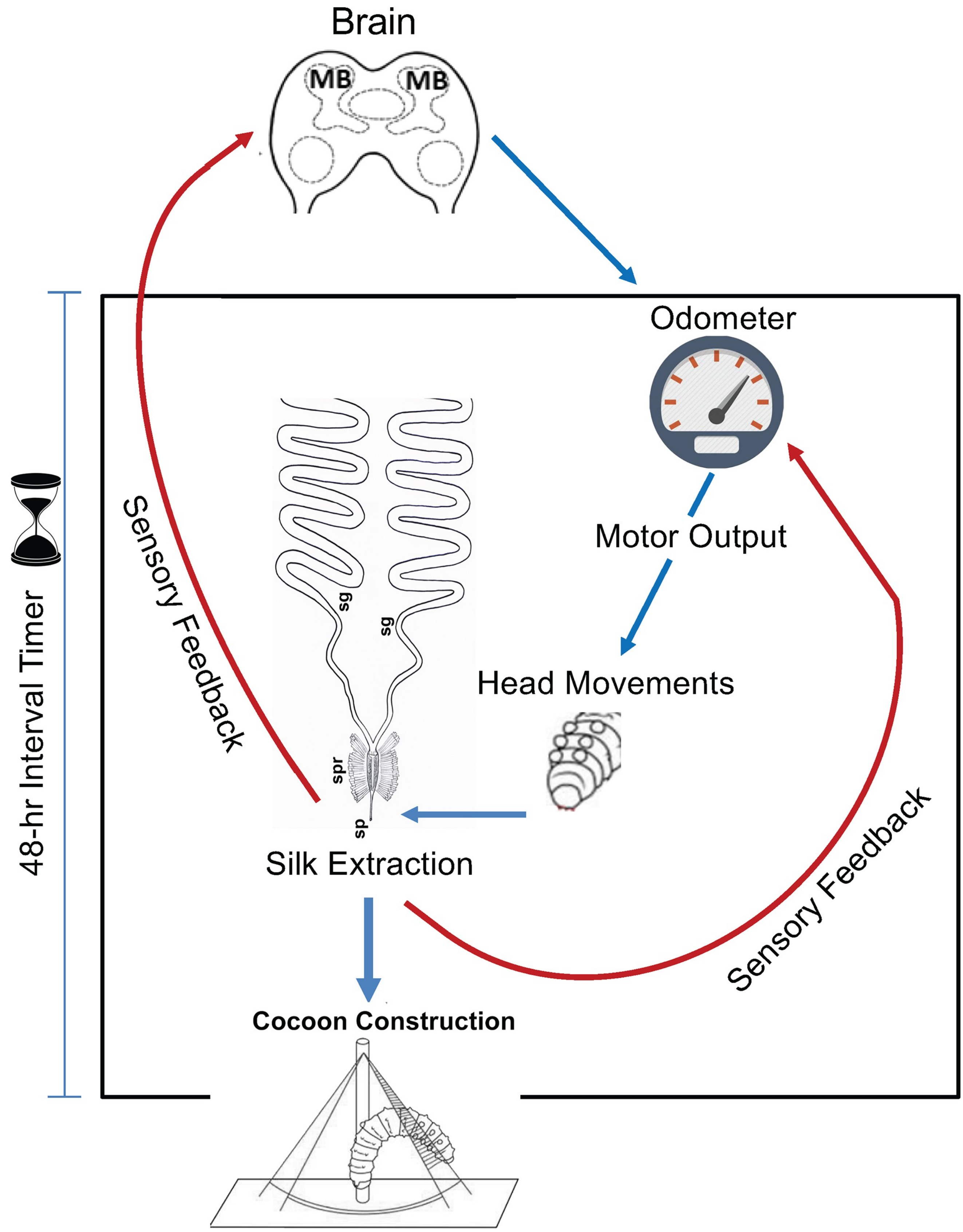
Brain
MB
MB
Odometer
Motor Output
Head Movements
Sensory Feedback
sg
sg
spr
sp
Silk Extraction
Sensory Feedback
48-hr Interval Timer
Cocoon Construction

Fig 10. Model of the cocoon construction program performed by the 5th instar cecropia caterpillar. We propose that the silk odometer resides in the pre-motor circuitry, ultimately controlled by the mushroom bodies (MBs) of the brain. The odometer regulates head movements which are necessary for silk extraction from the spinneret. Sensory information about silk length feeds back onto the odometer. This positive feedback loop is critical for driving the normal cocoon construction program, leading to the highly ordered execution of each of the discrete events of cocoon construction. The timing of the cocoon spinning program is limited to the 48-hr period defined by an interval timer (hourglass). If the timer is not activated soon after cocoon spinning normally begins, the construction program is disrupted. Abbreviations: silk gland (sg), silk press (spr), spigot (sp).

https://doi.org/10.1371/journal.pone.0228453.g010

with silk length measurement, will provide novel insights into the workings of an immature insect brain and its coordination of complex motor behavior.

Materials and methods

Animals

For studies in the United States, cocoons were collected in the field, at various locations characterized by new growth, mid- to low-lying shrubs found in disturbed areas along trails and power lines, and in successional habitats, in Central and Eastern Massachusetts (MA). Cecropia silkmoths are neither endangered nor protected, and no specific permits were required for collecting and using specimens in our study. Female moths that emerged from these field-collected cocoons called males from the wild and mated to generate caterpillars for cocoon spinning trials; >95% of laid eggs hatched. Caterpillars from the 1st instar to the 5th (final) instar were reared outdoors in mesh cages, with caterpillars fed fresh leaves cut from cherry trees (*Prunus*). Cocoons were spun under these conditions at Auburndale, Newton, MA USA (latitude 42˚ 20' N, longitude 71˚ 14' W). When a late 5th instar caterpillar had purged its gut, an indication of the onset of cocoon spinning, the time of gut purge was recorded and the animal was used in trials.

For studies in the Czech Republic, the eggs and pupae of cecropia were purchased from Worldwide Butterflies Ltd. (Dorset, England, www.wwb.co.uk). The larvae were reared in a standard insect rearing facility on freshly cut wild cherry leaves at 25˚C, relative humidity 60–80%, and light-dark cycle 16L:8D. Larva rearing and cocoon spinning occurred at Ceske Budejovice, Czech Republic (latitude 48˚ 58' N, longitude 14˚ 28'E).

Interruption of cocoon construction

To study the effect of an interruption of silk deposition during the cocoon construction program, we first allowed caterpillars to spin on branches contained in indoor mesh cages, and interrupted their cocoon spinning behavior by removing the caterpillar during the construction of the OE or after the OE was completed. Caterpillars were removed from the initial OE or completed OE by cutting open the cocoon fragment after being chilled for 30 min at 4˚C. After removal, each caterpillar was placed on a new branch in the mesh cage and allowed to continue spinning until completion of its cocoon.

To examine and compare the architectural properties of a caterpillar's initial OEs and 2nd spin OEs, we used a MakerBot Digitizer 3D scanner (MakerBot Industries) to obtain a 3D scan for each type of OE spun. Each OE 3D scan was then imported into the program netfabb Basic (Version 5.2.0, Autodesk, Inc.) to obtain surface area (SA) and volume (V) measurements. We compared the SA and V of initial and 2nd spin OEs (spring 2014 and 2016 trials) using paired t-tests. After completion of the 3D scans, we then weighed the silk (OE, IS, and IE) from a subset of cocoons that comprised the initial OE and the 2nd spin cocoons, compared to those from field-collected cocoons. Second, we removed caterpillars after the OE was impregnated, designating completion of the OE, and placed the caterpillars in a spinning arena to observe the spinning of the IE. All silk components were weighed using a balance

(Ohaus Aventurer Pro, Model AV313, Ohaus Corporation). We used unpaired t-tests to compare the amount of silk contained within cocoons spun by caterpillars whose behavior we interrupted, with the amount of silk contained in cocoons collected in the field.

Use of pre-spun cocoon envelopes

To determine whether or not caterpillars are required to spin specific sections of the cocoon during the cocoon construction process, we performed trials similar as those conducted by Van der Kloot & Williams [7]. Briefly, we placed a post-gut purge caterpillar in either a cut-open pre-spun OE that was spun on a branch, sewed the opening of the pre-spun OE with thread to fully contain the caterpillar, and allowed the animal to spin a cocoon until completion. At seven days after cocoon construction, we re-opened the pre-spun OE in order to examine the architecture of the cocoon spun by the caterpillar, e.g., examine for the number of cocoon layers that were spun. We also weighed the OEs spun by caterpillars and statistically compared their weights to the OEs of field-collected cocoons using an unpaired t-test. In another set of trials, we allowed caterpillars to spin an OE to completion (after impregnation), and once impregnated, we removed each caterpillar from its OE. After removal, we placed each caterpillar within an open pre-spun IE, sewed the pre-spun IE shut to fully contain the animal. At seven days after cocoon construction, we then examined the morphology of the cocoon spun by caterpillars within the pre-spun IE.

Blocking silk extraction

We also examined how the behavior of caterpillars would be altered if individuals were unable to extract silk as normal, i.e., control the flow of silk out of the spinneret, once the cocoon construction process had been initiated after gut-purge. Post-gut purge caterpillars were immediately brought into the laboratory and introduced into a 4˚C cold room to immobilize animals for spinneret blocking treatments. Once immobile, a caterpillar had the tip of its spinneret blocked with Loctite ultragel super glue (Henkel Corporation) to prevent the extraction of silk, and the animal was then left in the cold room for one hour for the glue to dry. For control caterpillars, after the glue was allowed to dry for one hour, the spigot of the spinneret with the super glue was severed using scissors to allow for the normal extraction of silk. For both treatment and control caterpillars, each animal was then introduced into its own spinning arena after this hour to allow for cocoon construction. A spinning arena consisted of a clear, plastic box (8.89 x 8.89 x 12.7 cm; US Acrylic), with a poplar dowel (length: 10.16 cm; diameter: 0.635 cm) positioned in the middle of the box to simulate a branch. The cocoon construction behavior of each animal in the spinning arena [10] was observed each day, on a regular basis during the trial period (e.g., every hour for about 10 minutes during the morning, afternoon, and early evening), during the animal's subjective day. Each animal was observed in order to record for any performance of wandering behavior and of the three behaviors related to cocoon construction (stretch-bend, swing-swing, figure-8). For all of these behaviors, if the animal was seen to exhibit the behavior, it was scored as having performed the specific behavior for that day. In addition to manual observation, each animal was continuously monitored via a camera (Pro 550, Swann Communications), with footage from the camera recorded onto a digital video recorder (H.264 DVR-8-2550 digital video recorder, Swann Communications). Twenty-four hours after the start of these cocoon-spinning trials, we then clipped the tip of the spinneret of treatment caterpillars to remove the super glue that blocked the spinneret opening. After clipping, we immediately returned treated caterpillars to the spinning arenas, and continued to monitor their behavior until the end of the trials.

Silk gland surgery

Silk gland surgery was performed under carbon dioxide anesthesia. To achieve continuous anesthesia, a caterpillar was placed into a small plastic bag with continuous carbon dioxide inflow and only a small opening in the wall of the bag was made in the place of the surgery. The cuticle was first sterilized by moistening with 96% ethanol and then cut with a fine micro-surgery scissors. Fine biological forceps were used to sever the silk gland at the desired position (Fig 5A). The incised cuticle was then stitched together with a surgical nylon thread and the area of the incision was sterilized with 96% ethanol to coagulate the hemolymph excurrent from the wound. After finishing spinning, the pre-pupae were removed from the completed cocoons and dissected under a microscope to confirm the appropriate parts of the silk glands were successfully ablated.

Sensilla extirpation

Last instar larvae were water-anesthetized by submerging in distilled water for approximately 15 min. One or both spinneret sensilla were completely removed under the stereomicroscope. In order to prevent hemolymph leakage, a thin layer of silicone grease was used to cover the wounds. Following the surgical treatment the animals were placed individually into empty transparent plastic boxes for cocoon spinning.

Whole-mount preparation of spinning apparatus

The spinning apparatus with the surrounding tissues were dissected from water-anaesthetized animals (submerged in sterile distilled water for approximately 15 min) under sterile 0.1M sodium phosphate buffer (PB), and fixed in 4% paraformaldehyde in PB for 2 h at room temperature (RT). Following the washes in PB (four times for 15 min at RT) tissues were treated with phalloidin conjugated with Alexa Fluor 448 (Invitrogen) diluted 1:200 in PB supplemented with 0.5% Triton X-100 (PB-T) for 10 min at RT. After a thorough rinsing with PB-T (four times for 15 min at RT), the preparations were stained with DAPI diluted in distilled water (1 μg/ml) for 15 min at RT and then washed in distilled water (three times for 15 min at RT). The tissues were dehydrated in ethanol series followed by incubation in 1:1 mixture of 100% ethanol and methyl salicylate. After evaporation of ethanol the tissues were mounted in methyl salicylate. The samples were examined under Olympus FV 1000 confocal microscope (Olympus) using correction of brightness in depth and multi-positional scanning. Images of the whole spinning apparatus were reconstructed by stitching of individual frames using Xuv-Stitch software (XuvTools). The compound images were then analyzed with Imaris image analysis software (Bitplane) using the following modules: Easy 3D and Surpass—Surfaces.

Neuronal tracing

A half of the spinneret (spigot) sensillum was cut off from water-anesthetized last instar larvae, and a drop of Alexa Fluor-conjugated dextran (A546 or A594, 10,000 MW, 10% solution in sterile distilled water) was applied on the incision by a glass microcapillary. The wound was covered by a thin layer of silicone grease to prevent drying after the application of the fluorescent tracers. Treated animals were kept on regular diet in constant darkness for two days. Subsequently spinneret with the attached sensilla together with complete cephalic ganglion was dissected and tissue processing was performed as described above for staining with phalloidin and DAPI.

Transmission electron microscopy

Dissected tissues from water anaesthetized last instar larvae were fixed in 2.5% glutaraldehyde in 0.1M PB for five days at 4˚C. The samples were then post-fixed in 2% osmium tetroxide in PB for 2 hr at 4˚C, dehydrated, and embedded in Epon 812 resin. Semi-thin sections (1 μm) were stained with toluidine blue. Ultrathin sections (70 nm) were contrasted with uranyl acetate and lead citrate, and examined with Jeol 1010 transmission electron microscope.

Scanning electron microscopy

The whole heads or dissected tissues were fixed in 2.5% glutaraldehyde in 0.1M PB for five days at 4˚C. The samples were washed in 0.1M PB supplemented with 4% glucose (three times for 15 min at RT) and subsequently treated with 2% osmium tetroxide in PB for 2 hrs at 4˚C. After dehydration and critical point drying the samples were sputter-coated with gold. The silk samples from the cocoons were directly gold coated without any previous treatment. All samples were viewed and photographed using Jeol JSM-7401F scanning electron microscope.

Supporting information

S1 Text. Spinneret details.
(PDF)

S2 Text. Spinneret comparison with other species.
(PDF)

S1 Fig. Interrupting silk deposition during baggy cocoon construction.
(PDF)

S2 Fig. The silk spinning apparatus region of cecropia.
(PDF)

S3 Fig. Transverse sections of the cecropia spinning apparatus (light microscopy images).
(PDF)

S1 Video. Spinning behavior of a cecropia caterpillar. The video shows a 5th instar caterpillar spinning the scaffold and outer envelope of a cocoon on a dowel in a spinning arena. It is a time-lapse view of the first 16 hrs of spinning behavior.
(MP4)

Acknowledgments

We thank David R. Weaver for logistical support.

Author Contributions

Conceptualization: Patrick A. Guerra, Ivo Sauman, Steven M. Reppert.

Data curation: Hana Sehadova, Patrick A. Guerra, Ivo Sauman, Steven M. Reppert.

Formal analysis: Hana Sehadova, Patrick A. Guerra, Ivo Sauman, Steven M. Reppert.

Funding acquisition: Patrick A. Guerra, Ivo Sauman, Steven M. Reppert.

Investigation: Hana Sehadova, Patrick A. Guerra, Ivo Sauman, Steven M. Reppert.

Methodology: Hana Sehadova, Patrick A. Guerra, Ivo Sauman, Steven M. Reppert.

Project administration: Patrick A. Guerra, Ivo Sauman, Steven M. Reppert.

Resources: Hana Sehadova, Patrick A. Guerra, Ivo Sauman, Steven M. Reppert.

Supervision: Patrick A. Guerra, Ivo Sauman, Steven M. Reppert.

Validation: Hana Sehadova, Patrick A. Guerra, Ivo Sauman, Steven M. Reppert.

Visualization: Hana Sehadova, Patrick A. Guerra, Ivo Sauman, Steven M. Reppert.

Writing – original draft: Hana Sehadova, Patrick A. Guerra, Ivo Sauman, Steven M. Reppert.

Writing – review & editing: Hana Sehadova, Patrick A. Guerra, Ivo Sauman, Steven M. Reppert.

References

1. Wolf H. Odometry and insect navigation. J Exp Biol. 2011; 214: 1629–1641. https://doi.org/10.1242/jeb.038570 PMID: 21525309
2. Wittlinger M, Wehner R, Wolf H. The ant odometer. Science 2006; 312: 1965–11967 https://doi.org/10.1126/science.1126912 PMID: 16809544
3. Wittlinger M, Wehner R, Wolf H. The desert ant odometer: a stride integrator that accounts for stride length and walking speed. J Exp Biol. 2007; 210: 198–207. https://doi.org/10.1242/jeb.02657 PMID: 17210957
4. Land MF, Stepping movements made by jumping spiders during turns mediated by the lateral eyes. J Exp Biol. 1972; 57: 15–40. PMID: 5075890
5. Walls ML, Layne JE. Direct evidence for distance measurement via flexible stride integration in the fiddler crab. Curr Biol. 2009; 19: 25–29. https://doi.org/10.1016/j.cub.2008.10.069 PMID: 19110426
6. Van der Kloot WG, Williams CM. Cocoon construction by the cecropia silkworm. I. The role of the external environment. Behaviour. 1953; 5: 141–156.
7. Van der Kloot WG, Williams CM. Cocoon construction by the cecropia silkworm. II. The role of the internal environment. Behaviour. 1953; 5: 157–174.
8. Van der Kloot WG, Williams CM. Cocoon construction by the cecropia silkworm. III. The alteration of spinning behavior by chemical and surgical techniques. Behaviour. 1954; 6: 233–255.
9. Waldbauer GP, Scarborough AG, Sternburg JG. The allocation of silk in the compact and baggy cocoons of *Hyalophora cecropia*. Entomol Exp Appl. 1982; 31: 191–196.
10. Guerra PA, Reppert SM. Dimorphic cocoons of the cecropia moth (*Hyalophora cecropia*): Morphological, behavioral, and biophysical differences. PLoS ONE. 2017; 12(3):e0174023. https://doi.org/10.1371/journal.pone.0174023 PMID: 28329006
11. Danks HV. Modification of adverse conditions by insects. Oikos. 2002; 99: 10–24.
12. Danks HV. The roles of insect cocoons in cold conditions. Eur J Entomol. 2004; 101: 433–437.
13. Lounibos L. Initiation and maintenance of cocoon spinning behaviour by saturniid silkworms. Physiol Entomol. 1976; 1: 195–206.
14. Scarborough AG, Sternburg JG, Waldbauer GP. Selection of the cocoon spinning site by the larvae of *Hyalophora cecropia* (Saterniidae). J Lep Soc. 1977; 31: 153–166.
15. Weevers R de G. The insect ganglia. In: Kerkut GA, Gilbert LI, editors. Comprehensive insect physiology biochemistry and pharmacology, vol. 5. New York: Pergamon Press; 1985. p. 213–297.
16. Zacharuk RY. Antennae and sensilla. In: Kerkut GA, Gilbert LI, editors. Comprehensive insect physiology biochemistry and pharmacology, vol. 6. New York: Pergamon Press; 1985.p. 1–69.
17. Berkowitz A. Expanding our horizons: central patter generation in the context of complex activity complexes. J Exp Biol. 2019;222
18. Tuskes PM, Collins ML, Tuttle JP. The wild silk moths of North America: a natural history of the Saturniidae of the United States and Canada. 1st ed. Ithaca, NY: Cornell University Press; 1996.
19. Lounibos LP. The cocoon spinning behavior of the Chinese oak silkworm, *Antheraea pernyi*. Anim Behav. 1975; 23: 843–853
20. Costa JT. Caterpillars as social insects. Am Sci. 1997; 85: 150–159.
21. Fitzgerald TD, Willer DE. Tent-building behavior of the Eastern tent caterpillar *Malacosoma americanum* (Lepidoptera: Lasiocampidae). J Kans Entomol Soc.1983; 56: 20–31.

22. Gromysz K. Research on the plasticity of building behavior in caterpillars of the bagworm *Psyche viciella* Schiff. Folia Biol.1960; 8: 351–415.
23. Merrill D. The stimulus for case-building activity in caddis-worms (*Trichoptera*). J exp Zool. 1956; 158: 123–132.
24. Edgerly JS, Davilla JA, Schoenfeld N. Silk spinning behavior and domicile construction in webspinners. J Insect Behav. 2002; 15: 219–242.
25. Reed CF. Cues in web-building process. Amer Zool.1969; 9: 211–221.
26. Hesselberg T. Exploration behaviour and behavioural flexibility in orb-web spiders: a review. Current Zoology. 2015; 61: 313–327.
27. Eberhard WG. Adaptive flexibility in cues guiding spider web construction and its possible implications for spider cognition. Behaviour. 2019; 156: 331–362.
28. Turner-Evans DB, Jayaraman V. The insect central complex. Curr Biol. 2016; 26: 453–457.
29. Huetteroth W, el Jundi B, el Jundi S, Schachtner J. 3D-reconstructions and virtual 4D-visualization to study metamorphic brain development in the sphinx moth *Manduca sexta*. Front Syst Neurosci. 2010; 4: 1–15. https://doi.org/10.3389/neuro.06.001.2010
30. Campbell RA, Turner GC. The mushroom body. Curr Biol. 2009; 20: 11–12.
31. Wahdepuhl H, Huber F. Elicitation of singing and courtship movements by electrical stimulation of the brain of the grasshopper. Naturwissenschafter. 1979; 66:320–322.
32. Martin J-R, Ernst R, Heisenberg M. Mushroom bodies suppress locomotor activity inf Drosophila melanogaster. Learn Mem. 1998; 5: 179–191. PMID: 10454382
33. Gronenberg W, Lopez-Riquelme GO. Multisensory convergence in the mushroom bodies of ants and bees. Acta Biol Hung. 2004; 55: 31–37. https://doi.org/10.1556/ABiol.55.2004.1-4.5 PMID: 15270216
34. Hiroi M, Ohkura M, Nakai J, Masuda N, Hashimoto K, Inoue K, et al. Principal component analysis of odor coding at the level of third-order olfactory neurons in Drosophila. Genes Cells. 2013; 18: 1070–1081. https://doi.org/10.1111/gtc.12094 PMID: 24118654
35. Skorupski P, MaBouDi H, Dona HSG, Chittka L. Counting insects. Phil Trans R Soc B. 2017; 373 (1740). pii: 20160513. https://doi.org/10.1098/rstb.2016.0513 PMID: 29292360
